# Coffee and cancer risk: A meta-analysis of prospective observational studies

**DOI:** 10.1038/srep33711

**Published:** 2016-09-26

**Authors:** Anqiang Wang, Shanshan Wang, Chengpei Zhu, Hanchun Huang, Liangcai Wu, Xueshuai Wan, Xiaobo Yang, Haohai Zhang, Ruoyu Miao, Lian He, Xinting Sang, Haitao Zhao

**Affiliations:** 1 Department of Liver Surgery, Peking Union Medical College Hospital, Chinese Academy of Medical Sciences and Peking Union Medical College, Beijing, China; 2 Department of General Surgery, Tianjin Third Central Hospital, Tianjin Institute of Hepatobiliary Disease, Tianjin Key Laboratory of Artificial Cell, Tianjin, China; 3 Liver Center and The Transplant Institute, Department of Medicine, Beth Israel Deaconess Medical Center, Harvard Medical School, Boston, MA, USA

## Abstract

Meta-analyses on coffee and cancer incidence mainly restricted to limited cancers. We carried out a more comprehensive meta-analysis of cohort studies to explore association between coffee and most cancer types. We conducted comprehensive search and summarized relative risk (RR) and 95% confidence intervals for the highest versus lowest coffee intake and cancer using STATA12. We conducted dose-analysis if result suggested significant association. The publication bias was evaluated with begg’s and egger’s test. Finally, 105 individual prospective studies were included. Inverse associations were observed on oral, pharyngeal, colon, liver, prostate, endometrial cancer and melanoma, with RR 0.69 (95% CI = 0.48–0.99, I^2^ = 73.4%, P = 0.044), 0.87 (95% CI = 0.78–0.96, I^2^ = 28.4%, P = 0.007), 0.46 (95% CI = 0.37–0.57, I^2^ = 0%, P = 0), 0.89 (95% CI = 0.84–0.93, I^2^ = 30.3%, P = 0.003), 0.73 (95% CI = 0.67–0.80, I^2^  = 0%, P = 0) and 0.89 (95% CI = 0.80–0.99, I^2^  = 0%, P = 0.031) respectively. However, the relative risk for lung cancer is 2.18 (95% CI = 1.26–3.75, I^2^  = 63.3%, P = 0.005). The summary relative risk for increment of 2 cups of coffee were RR = 0.73, 95% CI = 0.67–0.79 for liver cancer, RR = 0.97, 95% CI = 0.96–0.98 for prostate cancer and RR = 0.88, 95% CI = 0.85–0.92 for endometrial cancer. Accordingly, coffee intake was associated with reduced risk of oral, pharynx, liver, colon, prostate, endometrial cancer and melanoma and increased lung cancer risk.

Cancer is a major cause of morbidity and mortality worldwide. In 2012^1^,^2^, there were 14.1 million new cancer cases and 8.2 million cancer deaths globally and the global burden from cancer has become significantly high. Although the strategy for cancer treatment has improved much, cancer is still the most difficult disease to overcome. Early diagnosis and treatment may provide good chance for cancer patients to improve overall prognosis. However, there is no promising survival for those who suffer from advanced cancer. Therefore, it is important to determine methods to prevent tumor occurrence. Nutrition, such as the consumption of citrus fruits, vegetables, and coffee, has also been considered to play an important role in reducing cancer risk^3^,^4^,^5^.

Coffee is one of the most popular beverages worldwide, and it has been speculated to decrease the risk of many types of cancers. Coffee is a complex mixture of many biologically active components, some of which may have anti-tumor effects. They include caffeine, cafestol, kahweol, and chlorogenic acid. Although many standardized meta-analyses^5^,^6^,^7^,^8^,^9^,^10^,^11^,^12^,^13^,^14^,^15^,^16^,^17^,^18^ between coffee intake and various cancers have been conducted, most of analyses restricted to limited types of cancers. In addition, there is no relevant meta-analysis to explore relationships between coffee intake and some types of cancer including melanoma and lymphoma. Furthermore, dose-response meta-analyses were only performed for liver cancer and prostate cancer^19^,^20^, we endeavor to conduct such analysis for cancer as many as possible. Besides, most meta-analyses included prospective studies and case-control studies, which may lead to the unconvincing results and conclusions. However, we included prospective studies into our study. In order to present the relatively obvious associations between coffee intake and the incidence of most of cancer types, we carried out a more comprehensive systematic updated meta-analysis of cohort studies to explore the association between coffee consumption and most types of cancers. We try to provide a landscape of coffee and cancer incidence.

## Methods

### Search strategy

**Figure 1:**
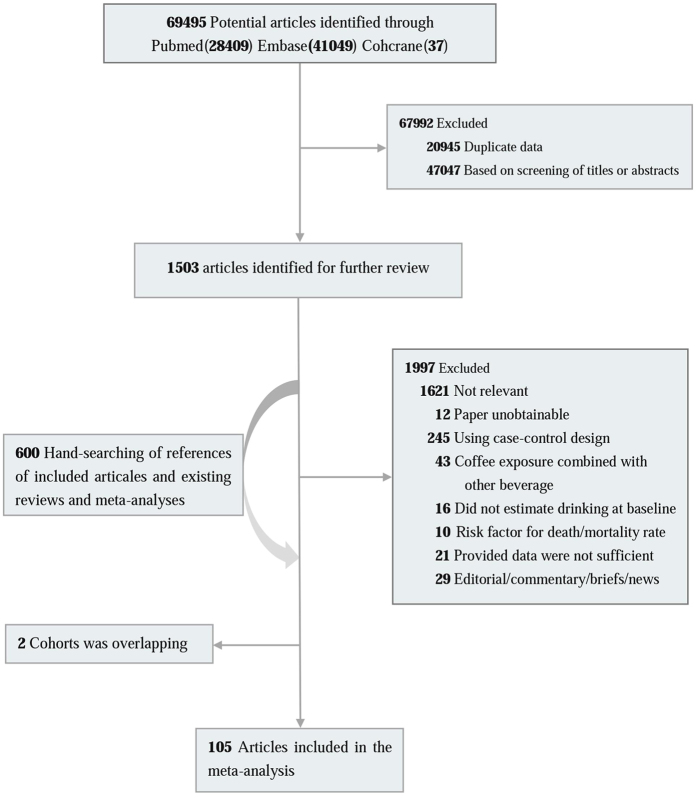
Flowchart of the searching and review of literatures.

We conducted a computerized search of the literature on coffee and cancer from inception to July 2015. The search terms were (cancer OR tumor OR carcinoma) AND (coffee OR caffeine OR beverages OR diet OR drinking). Three major electronic databases (PubMed, EMBASE, and The Cochrane Library) were used to search the relevant literature without language restriction. Then, we classified these studies into different groups according to cancer type and conducted repeated searches. Finally, we obtained a relatively complete literature record for every type of cancer. Moreover, we reviewed the references from retrieved articles for additional studies (Fig. 1).

### Study selection

The included studies had to be prospective cohort, case-cohort, or nested case control studies and had to contain the association between coffee intake and cancer incidence. Case control studies and cross-sectional studies were excluded. Studies concerning the coffee intake and the mortality of cancer were also excluded. We also excluded the analysis about cancer if there was no enough to conduct meta-analysis or no updated prospective studies for previous meta-analysis. Each study had to provide estimates of hazard ratio (HR) or relative risk (RR) with 95% confidence intervals or numbers of cases and controls to calculate those values. For cancers for which a dose-response analysis could be conducted, the quantitative measure of intake and the total number of cases and person years or relative information to calculate them were necessary. Two independent reviewers evaluated the eligibility of every study in a standardized manner. We resolved disagreements by consensus.

### Data abstraction

We extracted data from each study. These data included the first author’s name, year of publication, country of origin, number of cases and participants, sex, age, types of cancer, duration of follow-up and comparison of exposure level and adjusted confounding variables. The relative risks or hazard ratio and 95% confidence intervals for the highest versus the lowest intake were also extracted.

### Statistical analysis

We summarized the overall relative risk and 95% confidence intervals for the highest versus lowest intake of coffee and various cancers using the fixed or random effects models of STATA12 (StataCorp, College Station, TX, USA). The command is metan logrr loglci loguci, eform label(namevar = study) fixed/random xlabel(), effect(RR). Metareg logrr study, wsse(se). The fixed and random effects models were conducted when there were no or low heterogeneity and medium or high heterogeneity, respectively. For studies that reported results by cancer type or sex, we combined them using fixed effects models. For studies that provided no relative risks and 95% confidence intervals, we calculated these values based on the number of cases and controls.

We conducted dose-response analyses for coffee and some types of cancers rather than all tumor types. When the summary RR indicates a significant association between coffee intake and cancer risk, we would conduct dose-response analysis for these types of cancers. Furthermore, the included studies for dose-response analysis had to be three at least. According to a previously described method^21^,^22^, information was required, such as the distribution of cases and person-years, variance of exposure levels and relative risks for at least three quantitative categories. The number of person-years was approximated from follow-up duration and number of subjects if the studies did not provide these values. We assigned the mid-point of the corresponding range of coffee intake as the exposure value in each category. When the highest category was open-ended, we assumed the width of the interval to be the same as the adjacent interval. When the lowest category was open-ended, we assigned zero as the lower boundary^22^. If coffee intake was reported as weeks or months instead of days, we computed the corresponding days. If coffee intake was not measured in cups, we used 150 ml^23^ as a cup to recalculate the intakes to a common scale. We present the dose-response results for a two-cup daily increment of coffee consumption. Using restricted cubic splines with four knots at percentiles 5%, 35%, 65% and 95% of the distribution; we evaluated the curve linear relation between coffee intake and risks of liver cancer, prostate cancer and endometrial cancer. The P value was calculated to test the null hypothesis that the coefficients of the second and third splines are equal to zero^24^,^25^. According to the result, we conducted linear or non-linear dose-response analysis.

We used the I^2^ value to evaluate if heterogeneity could be explained by study differences rather than by chance^26^. I^2^-values of approximately 25%, 50%, and 75% indicate low, moderate and high heterogeneity. We did not use scores to assess the quality of the studies. Instead, we conducted subgroup analyses to evaluate the impact on overall RRs by cancer type, sex, follow-up period and some adjusted confounders, such as alcohol and BMI. Moreover, when the number of included studies was more than 10 and a substantial heterogeneity was observed, we would conduct subgroup analysis and meta-regression analysis to explore the potential source of heterogeneity. Furthermore, when the number of included studies was more than 10 and a substantial heterogeneity was observed, we would conduct subgroup analysis and meta-regression analysis to explore the potential source of heterogeneity.

We evaluated publication bias using Begg’s^27^ and Egger’s^28^ tests, with obvious publication bias suspected when P < 0.10. And we conducted a Trim and fill analysis^29^ to assess the stability of overall relative risk when the results suggested obvious publication bias. We conducted all of the statistical analyses using Stata Statistical Software, version 12.0.

## Results

### Digestive system cancer

#### Oral, pharynx cancer

**Figure 2:**
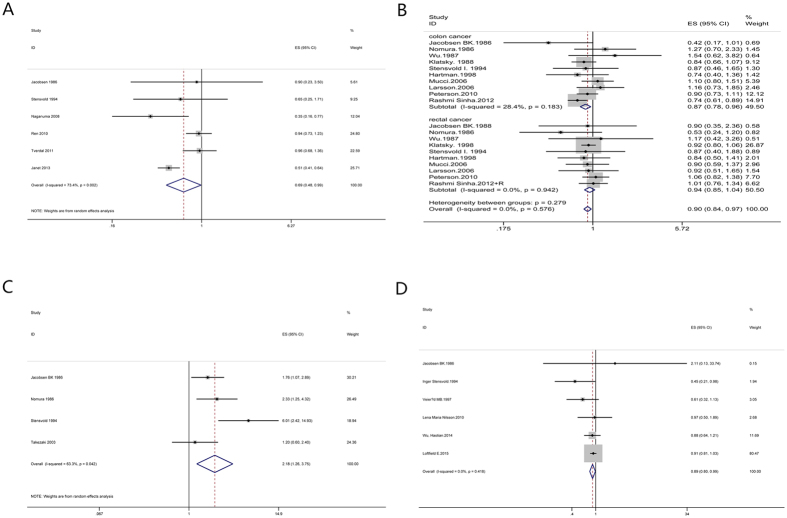
Meta-analyses between coffee intake and risk of oral, pharynx cancer, colorectal cancer, lung cancer and melanoma. Relative risks of oral, pharynx cancer (**A**), colorectal cancer (**B**), lung cancer (**C**) and melanoma (**D**) associated with coffee intake. Squares represent study-specific relative risk estimates (size of the square reflects the study-specific statistical weigh, that is, the inverse of the variance); horizontal lines represent 95% CIs; diamonds represent summary relative risk estimates with corresponding 95% CIs.

Highest versus lowest intake: Six cohort studies^30^,^31^,^32^,^33^,^34^,^35^ were included in the analysis (1395309 samples) of the highest versus lowest intake of coffee and oral, pharynx cancer. The study characteristics are presented (Stable 1a). The summary RR was 0.69 (95% CI = 0.48–0.99, P = 0.044) with high heterogeneity (I^2^ = 73.4%, P = 0.002) (Fig. 2A). The results suggest no publication bias, with P = 1 for Begg’s test and P = 0.98 for Egger’s test. The subgroup analysis indicated that the inverse association was observed between coffee consumption and oral, pharynx cancer incidence in Asia and follow-up (>10 years) subgroup. The same relationship was observed in smoking adjustment subgroup, physical activity adjustment subgroup and total energy adjustment subgroup (Stable 1b).

#### Esophageal cancer

**Figure 3:**
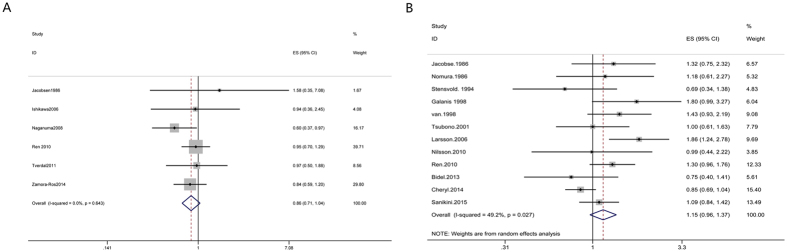
Meta-analyses between coffee intake and risk of esophageal cancer and stomach cancer. Relative risks of esophageal cancer (**A**) and stomach cancer (**B**) associated with coffee intake. Squares represent study-specific relative risk estimates (size of the square reflects the study-specific statistical weigh, that is, the inverse of the variance); horizontal lines represent 95% CIs; diamonds represent summary relative risk estimates with corresponding 95% CIs.

Highest versus lowest intake: Six cohort studies^30^,^32^,^33^,^34^,^36^,^37^ were included in the analysis (1395309 samples) of the highest versus lowest intake of coffee and esophageal cancer. The study characteristics are presented (Stable 1a). The summary RR was 0.86 (95% CI = 0.71–1.04, P = 0.124) with no heterogeneity (I^2^ = 0%, P = 0.64) (Fig. 3A). The results suggest no publication bias, with P = 1 for Begg’s test and P = 0.69 for Egger’s test. The subgroup analysis suggested that no significant association was observed between coffee intake and esophageal cancer incidence in each subgroup (Stable 1b).

#### Stomach cancer

Highest versus lowest intake: Twelve cohort studies^30^,^31^,^33^,^38^,^39^,^40^,^41^,^42^,^43^,^44^,^45^,^46^ were included in the analysis (1305447 samples) of the highest versus lowest intake of coffee and stomach cancer. The study characteristics are presented (Stable 1a). The summary RR was 1.15 (95% CI = 0.96–1.37, P = 0.121) with medium heterogeneity (I^2^ = 49.2%, P = 0.027) (Fig. 3B). The results suggest no publication bias, with P = 0.63 for Begg’s test and P = 0.85 for Egger’s test. The subgroup analysis indicated that coffee intake was associated with increased stomach cancer in USA and Asia. The same relationship was also observed in small number cases subgroup (<500 cases) and short follow-up subgroup (<10 years). The meta-regression analysis found no obvious to explain source of heterogeneity (Stable 1b).

#### Colorectal cancer

**Figure 4:**
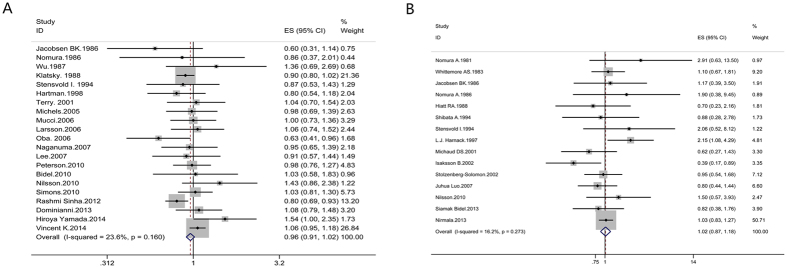
Meta-analyses between coffee intake and risk of colorectal cancer and pancreatic cancer. Relative risks of colorectal cancer (**A**) and pancreatic cancer (**B**) associated with coffee intake. Squares represent study-specific relative risk estimates (size of the square reflects the study-specific statistical weigh, that is, the inverse of the variance); horizontal lines represent 95% CIs; diamonds represent summary relative risk estimates with corresponding 95% CIs.

Highest versus lowest intake: Twenty-one cohort studies^30^,^31^,^38^,^43^,^47^,^48^,^49^,^50^,^51^,^52^,^53^,^54^,^55^,^56^,^57^,^58^,^59^,^60^,^61^,^62^,^63^ were included in the analysis (2141185 samples) of the highest versus lowest intake of coffee and colorectal cancer. The study characteristics are presented (Stable 1a). The summary RR was 0.96 (95% CI = 0.91–1.02, P = 0.175) with low heterogeneity (I^2^ = 23.6%, P = 0.160) (Fig. 4A). The summary RRs for colon and rectal cancer were 0.87 (95% CI = 0.78–0.96, P = 0.007) and 0.94 (95% CI = 0.85–1.04, P = 0.236) (Fig. 2B). The results suggest no publication bias, with P = 0.70 for Begg’s test and P = 0.82 for Egger’s test. The subgroup analysis indicated that no significant association was observed between coffee intake and the risk of colorectal cancer in each subgroup. No substantial source of heterogeneity was found by meta-regression analysis (Stable 1b).

#### Pancreatic cancer

Highest versus lowest intake: Fifteen cohort studies^30^,^31^,^38^,^44^,^64^,^65^,^66^,^67^,^68^,^69^,^70^,^71^,^72^,^73^,^74^ were included in the analysis (1219019 samples) of the highest versus lowest intake of coffee and pancreatic cancer. The study characteristics are presented (Stable 1a). The summary RR was 1.02 (95% CI = 0.87–1.18, P = 0.832) with low heterogeneity (I^2^ = 16.2%, P = 0.27) (Fig. 4B). The results suggest no publication bias, with P = 0.43 for Begg’s test and P = 0.75 for Egger’s test. There was no significant association between coffee intake and the risk of pancreatic cancer in each subgroup through subgroup analysis. Meta-regression analysis found no factors explaining the source of heterogeneity (Stable 1b).

#### Liver cancer

**Figure 5:**
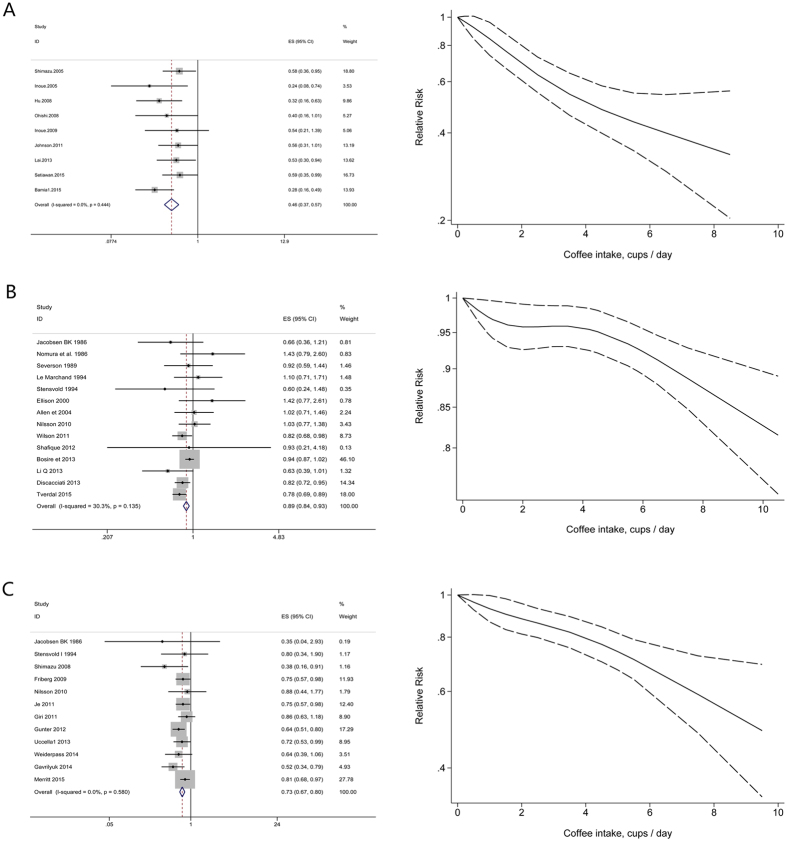
Dose-response analyses between coffee intake and risk of liver cancer, prostate cancer and endometrial cancer. Relative risks of liver cancer (**A**), prostate cancer (**B**) and endometrial cancer (**C**) associated with coffee intake. Squares represent study-specific relative risk estimates (size of the square reflects the study-specific statistical weigh, that is, the inverse of the variance); horizontal lines represent 95% CIs; diamonds represent summary relative risk estimates with corresponding 95% CIs.

Highest versus lowest intake: Nine cohort studies^23^,^75^,^76^,^77^,^78^,^79^,^80^,^81^,^82^ were included in the analysis (968517 samples) of the highest versus lowest intake of coffee and liver cancer. The study characteristics are presented (Stable 1a). The summary RR was 0.46 (95% CI = 0.37–0.57, P = 0) with no heterogeneity (I^2^ = 0%, P = 0.44) (Fig. 5A). The results suggest no publication bias, with P = 0.18 for Begg’s test and P = 0.23 for Egger’s test. The inverse association was observed between coffee consumption and liver cancer incidence in most subgroups through subgroup analysis. However, there was no significant relationship between coffee intake and the risk of liver cancer in women subgroup (Stable 1b).

#### Dose-response analysis

We included seven studies^23^,^75^,^76^,^77^,^78^,^79^,^80^,^82^ for the dose-response analysis. There was no non-linear association between coffee intake and risk of liver cancer (P = 0.41 for non-linearity, Fig. 5A). A statistically significant inverse association was observed for liver cancer (RR = 0.73, 95% CI = 0.67–0.79) with an increased intake of 2 cups of coffee per day.

### Tumors of the urinary system

#### Renal cancer

**Figure 6:**
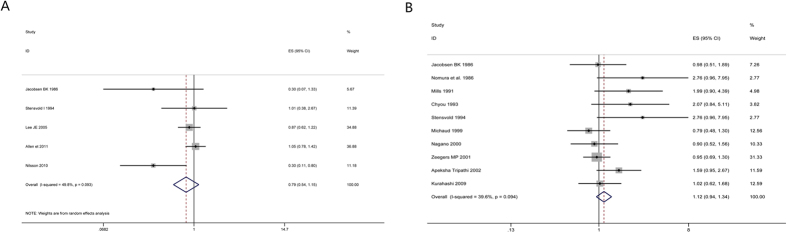
Meta-analyses between coffee intake and risk of renal cancer and bladder cancer. Relative risks of renal cancer (**A**) and bladder cancer (**B**) associated with coffee intake. Squares represent study-specific relative risk estimates (size of the square reflects the study-specific statistical weigh, that is, the inverse of the variance); horizontal lines represent 95% CIs; diamonds represent summary relative risk estimates with corresponding 95% CIs.

Highest versus lowest intake: Five cohort studies^30^,^31^,^43^,^83^,^84^ were included in the analysis (1036465 samples) of the highest group (samples/cases: 125065/144) versus lowest group (samples/cases: 232706/299) of coffee intake and renal cancer. The study characteristics are presented (Stable 2a). The summary RR was 0.79 (95% CI = 0.54–1.15, P = 0.226) with medium heterogeneity (I^2^ = 49.8%, P = 0.09) (Fig. 6A). The results suggest no publication bias, with P = 0.22 for Begg’s test and P = 0.14 for Egger’s test. There was no significant association between coffee intake and renal cancer risk in each subgroup through subgroup analysis (Stable 2bM). We did not found factors to explain the source of heterogeneity through subgroup analysis.

#### Bladder cancer

Highest versus lowest intake: Ten cohort studies^30^,^31^,^38^,^85^,^86^,^87^,^88^,^89^,^90^,^91^ were included in the analysis (340544 samples) of the highest versus lowest intake of coffee and bladder cancer. The study characteristics are presented (Stable 2a). The summary RR was 1.12 (95% CI = 0.94–1.34, P = 0.192) with medium heterogeneity (I^2^ = 39.6%, P = 0.094) (Fig. 6B). The results suggest publication bias, with P = 0.016 for Begg’s test and P = 0.01 for Egger’s test. The summary RR was 1.04 (95% CI = 0.88–1.23, P = 0.669) after Trim and fill analysis. The subgroup analysis indicated that coffee intake was associated with increased bladder cancer in USA (Stable 2b). No factor could explain the source of heterogeneity through subgroup analysis and meta-regression analysis (Stable 2b).

#### Prostate cancer

Highest versus lowest intake: Fourteen cohort studies^30^,^31^,^38^,^43^,^92^,^93^,^94^,^95^,^96^,^97^,^98^,^99^,^100^,^101^ were included in the analysis (864012 samples) of the highest versus lowest intake of coffee and prostate cancer. The study characteristics are presented (Stable 2a). The summary RR was 0.89 (95% CI = 0.84–0.93, P = 0.003) with medium heterogeneity (I^2^ = 30.3%, P = 0.14) (Fig. 5B). The results suggest no publication bias, with P = 0.83 for Begg’s test and P = 0.84 for Egger’s test. Through subgroup analysis, we found the inverse association between coffee intake and prostate cancer incidence in most subgroups. However, there was no significant relationship between coffee intake and the risk of prostate cancer in Canada and Asia (Stable 2b). The same associations were observed in follow-up (<10 years) subgroup and some adjustment confounders including no physical activity adjustment subgroup and no BMI adjustment subgroup (Stable 2b). Meta-regression found no substantial factor to explain the source of heterogeneity.

Dose-response analysis: We included ten studies^30^,^31^,^92^,^94^,^96^,^97^,^98^,^99^,^100^,^101^ in the dose-response analysis. There was no non-linear association between coffee intake and risk of prostate cancer (P = 0.15 for non-linearity, Fig. 5B). A statistically significant inverse association was observed for prostate cancer (RR = 0.97, 95% CI = 0.96–0.98) with an increased intake of 2 cups of coffee per day.

### Female genital system neoplasm

#### Breast cancer

**Figure 7:**
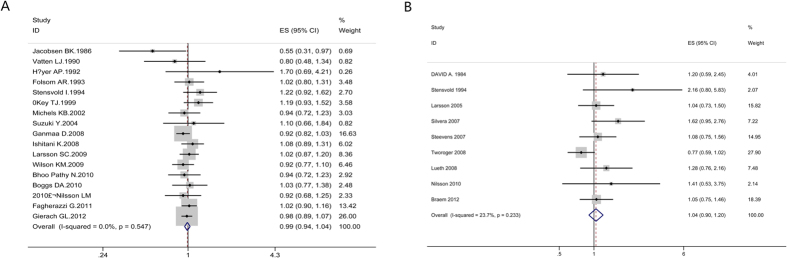
Meta-analyses between coffee intake and risk of breast cancer and ovarian cancer. Relative risks of breast cancer (**A**) and ovarian cancer (**B**) associated with coffee intake. Squares represent study-specific relative risk estimates (size of the square reflects the study-specific statistical weigh, that is, the inverse of the variance); horizontal lines represent 95% CIs; diamonds represent summary relative risk estimates with corresponding 95% CIs.

Highest versus lowest intake: Seventeen cohort studies^30^,^31^,^43^,^102^,^103^,^104^,^105^,^106^,^107^,^108^,^109^,^110^,^111^,^112^,^113^,^114^,^115^ were included in the analysis (997482 samples) of the highest versus lowest intake of coffee and breast cancer. The study characteristics are presented (Stable 3a). The summary RR was 0.99 (95% CI = 0.94–1.04, P = 0.619) with no heterogeneity (I^2^ = 0%, P = 0.55) (Fig. 7A). The results suggest no publication bias, with P = 0.84 for Begg’s test and P = 0.75 for Egger’s test. No significant relationship was observed between coffee consumption and the risk of breast cancer in all subgroups through subgroup analysis (Stable 3b).

#### Ovarian cancer

Highest versus lowest intake: Nine cohort studies^9^,^31^,^43^,^116^,^117^,^118^,^119^,^120^,^121^ were included in the analysis (687017 samples) of the highest versus lowest intake of coffee and ovarian cancer. The study characteristics are presented (Stable 3a). The summary RR was 1.04 (95% CI = 0.90–1.20, P = 0.582) with low heterogeneity (I^2^ = 23.7%, P = 0.23) (Fig. 7B). The results suggest publication bias, with P = 0.02 for Begg’s test and P = 0.009 for Egger’s test. The summary RR was 0.96 (95% CI = 0.84–1.09) after Trim and fill analysis. The subgroup analysis indicated that there was no significant association between coffee intake and ovarian cancer risk in each subgroup. No factor could explain the source of heterogeneity through subgroup analysis (Stable 3b).

#### Endometrial cancer

Highest versus lowest intake: Twelve cohort studies^30^,^31^,^43^,^122^,^123^,^124^,^125^,^126^,^127^,^128^,^129^,^130^ were included in the analysis (1114002 samples) of the highest versus lowest intake of coffee and endometrial cancer. The study characteristics are presented (Stable 3a). The summary RR was 0.73 (95% CI = 0.67–0.80, P = 0) with no heterogeneity (I^2^ = 0%, P = 0.58) (Fig. 5C). The results suggest no publication bias, with P = 0.19 for Begg’s test and P = 0.16 for Egger’s test. Subgroup analysis indicated that inverse significant association was observed between coffee consumption and endometrial cancer incidence in most subgroups. However, there was no significant relationship between coffee intake and the risk of endometrial cancer in no BMI adjustment subgroup (Stable 3b).

Dose-response analysis: We included eleven studies^30^,^31^,^43^,^122^,^123^,^124^,^125^,^126^,^127^,^128^,^129^ for the dose-response analysis. There was no non-linear association between coffee intake and risk of endometrial cancer (P = 0.69 for non-linearity, Fig. 5C). A statistically significant inverse association was observed for endometrial cancer (RR = 0.88, 95% CI = 0.85–0.92) with an increased intake of 2 cups of coffee per day.

### Other cancers

#### Lung cancer

Highest versus lowest intake: Four cohort studies^30^,^31^,^38^,^131^ were included in the analysis (103137 samples) of the highest versus lowest intake of coffee and lung cancer. The study characteristics are presented (Stable 4a). The summary RR was 2.18 (95% CI = 1.26–3.75, P = 0.005) with high heterogeneity (I^2^ = 63.3%, P = 0.04) (Fig. 2C). The results suggest no publication bias, with P = 0.73 for Begg’s test and P = 0.39 for Egger’s test.

#### Melanoma

Highest versus lowest intake: Six cohort studies^30^,^31^,^43^,^132^,^133^,^134^ were included in the analysis (773536 samples) of the highest versus lowest intake of coffee and melanoma. The study characteristics are presented (Stable 4a). The summary RR was 0.89 (95% CI = 0.80–0.99, P = 0.031) with no heterogeneity (I^2^ = 0%, P = 0.42) (Fig. 2D). The results suggest no publication bias, with P = 0.71 for Begg’s test and P = 0.40 for Egger’s test. There was no significant association between coffee intake and melanoma risk through subgroup analysis. The subgroup analysis indicated no factor to explain the source of heterogeneity (Stable 4b).

#### Lymphoma

**Figure 8:**
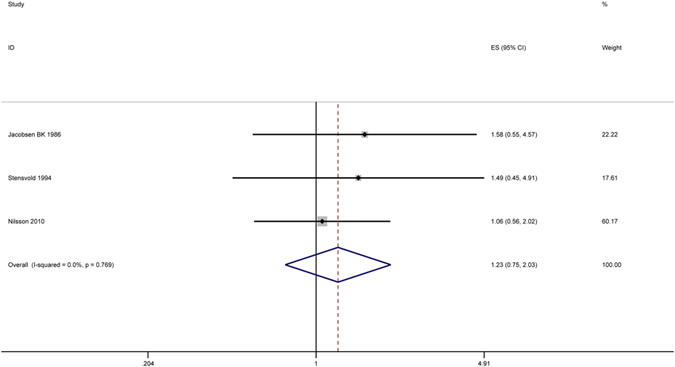
Meta-analyses between coffee intake and risk of lymphoma. Relative risks of lymphoma associated with coffee intake. Squares represent study-specific relative risk estimates (size of the square reflects the study-specific statistical weigh, that is, the inverse of the variance); horizontal lines represent 95% CIs; diamonds represent summary relative risk estimates with corresponding 95% CIs.

Highest versus lowest intake: Three cohort studies^30^,^31^,^43^ were included in the analysis (89897 samples) of the highest group (samples/cases: 32783/63) versus lowest group (samples/cases: 17229/22) coffee intake and lymphoma. The study characteristics are presented (Stable 4a). The summary RR was 1.23 (95% CI = 0.75–2.03, P = 0.415) with no heterogeneity (I^2^ = 0%, P = 0.769) (Fig. 8). The results suggest no publication bias, with P = 1 for Begg’s test and P = 0.18 for Egger’s test.

Subgroup, sensitivity, and meta-regression analyses: We conducted subgroup analyses and meta-regression analyses based on sex, duration of follow-up, geographical location and adjusted confounders. Coffee intake was associated with a decreased risk of liver cancer in most subgroups, with no substantial heterogeneity between subgroups. However, the protective effect was absent in the female population (Stable 1b). Inverse association was observed for oral, pharynx cancer in Asia rather than Europe and USA (Stable 1b). Inverse associations were also observed for prostate cancer and endometrial cancer, with no substantial heterogeneity between subgroups (Stables 2b and 3b). Coffee intake was not significantly associated with colorectal cancer incidence in most subgroup analyses. However, an inverse relation was observed for colon cancer (Stable 1b). An increased risk was observed for lung cancer in most subgroups (Stable 4b). No association was observed for esophageal cancer, stomach cancer, pancreatic cancer, renal cancer, bladder cancer, breast cancer, ovarian cancer or melanoma in subgroup analyses (Stables 1b, 2b, 3b and 4b).

We conducted a sensitivity analysis by excluding studies that were not dose-response analyses to evaluate whether the results were stable. The summary relative risk for the highest versus lowest intake of coffee and liver cancer was 0.53 (95% CI = 0.43–0.65, I^2^ = 0%, P = 0.693). The results were 0.87 (95% CI = 0.82–0.92, I^2^ = 31.8%, P = 0.154), 0.70 (95% CI = 0.63–0.78, I^2^ = 0%, P = 0.663), 0.85 (95% CI = 0.71–1.03, I^2^ = 05%, P = 0.396), 0.62 (95% CI = 0.41–0.94, I^2^ = 64.3%, P = 0.024) and 0.89 (95% CI = 0.72–1.11, I^2^ = 0%, P = 0.882) for prostate cancer, endometrial cancer, colon cancer, oral, pharynx cancer and melanoma. The results were similar to those of the primary analysis except for colon cancer and melanoma.

## Discussion

Our meta-analysis supports an inverse association between coffee intake and oral, pharynx cancer, liver cancer, colon cancer, prostate cancer, endometrial cancer and melanoma and increased association for lung cancer. Besides, the linear inverse associations were observed for liver cancer, prostate cancer and endometrial cancer. However, no significant association was found with esophageal cancer, stomach cancer, rectal cancer, pancreatic cancer, renal cancer, bladder cancer, prostate cancer, breast cancer, ovarian cancer, lung cancer, melanoma, and lymphoma.

To date, our study is the most comprehensive to conduct meta-analyses on coffee intake and cancer. We can clearly determine the association between coffee and most types of cancers from our research. Our findings seem to be more stable because our analyses were based on prospective studies. We conducted subgroup analyses and sensitivity analyses to explore the source of heterogeneity and verify the stability of the results. We also quantified the association between coffee intake and liver cancer, prostate cancer and endometrial cancer by conducting linear and non-linear dose-response analyses. Begg’s and Egger’s methods were used to evaluate publication bias. When the results suggested publication bias, we conducted a trim and fill analysis to determine whether the results were different.

Some limitations of our study should be taken into consideration. First, not all of the included studies conducted analyses by potential confounding, which could affect the results. However, we conducted subgroup analyses by confounding factors and found no substantial factors influencing the results. Second, some eligible studies^30^,^38^,^64^ did not provide relative risks and confidence intervals; thus, we extracted raw data for further analysis, which may influence the accuracy of the overall results regardless of confounding factors. Third, some studies measured the level of coffee intake in milliliters and others reported as cups. Due to the ambiguity in the volume contained in a cup of coffee, it was difficult to precisely compare the amount of coffee intake, which could influence the results of dose-response analyses. Moreover, we excluded a few studies for dose-response analyses. However, the results are similar to those of the analyses of highest versus lowest intake when we conducted sensitivity analyses by excluding these same data. Fourth, some cancers such as oral, pharynx cancer, esophageal cancer, renal cancer, lung cancer, melanoma and lymphoma included relatively limited number of studies for meta-analysis, may cause problems for evaluation of heterogeneities and publication bias and finally reduce the confidence of the results. Finally, our study did not include all types of cancers such as skin cancer, laryngeal carcinoma and glioma.

In addition, we found high heterogeneities when we conducted meta-analyses on coffee intake and oral, pharynx cancer and lung cancer. Although an inverse association was found between coffee and oral and pharynx cancer, we did not conduct a further dose-response analysis because of substantial heterogeneity. The dose-response analyses were also not conducted for colon cancer, melanoma and lung cancer because of the null results of sensitivity analyses. Medium heterogeneities were found in the analyses of coffee intake and renal cancer, bladder cancer and stomach cancer. Heterogeneity refers to the inconsistency within included studies. The biology diversity and methodological diversity could bring about heterogeneity. To further explore the source of heterogeneity, we conducted subgroups analyses and meta-regression analyses based on many factors, such as cancer subtypes, geographical location, sex, duration of follow-up and potential confounding factors^135^. We found publication bias in meta-analyses on coffee intake and liver cancer, bladder cancer and ovarian cancer. We conducted trim and fill analyses and found that all of the results were stable. However, the evaluation for publication bias is not very reliable when the number of include studies was small. Therefore, we could not draw conclusion that there was no obvious publication bias for esophageal cancer, renal cancer, melanoma and lymphoma.

Coffee intake could reduce the incidence of colon cancer, liver cancer, prostate cancer, endometrial cancer, oral, pharyngeal cancer and melanoma. There are several mechanisms attempting to explain this phenomenon. Coffee contains many bioactive components, including caffeine, cafestol, kahweol, and chlorogenic acid. Some studies indicate that caffeine can prevent oxidative DNA damage, modify the apoptotic response and reverse the cell cycle checkpoint function^136^,^137^. Moreover, some researchers^138^,^139^ have found that cafestol and kahweol are anticarcinogenic. Feng *et al*. hypothesized that chlorogenic acids can clear away reactive oxygen species and confer an anti-tumor effect^140^. Dong *et al*. demonstrated that caffeine suppresses the progression of HCC through the Akt signaling pathway^141^. Furthermore, caffeine and other compounds in coffee increased the clearance of estradiol and inhibited estradiol-mediated carcinogenesis in endometrial cells^142^. Coffee consumption decreased the exposure of epithelial cells to carcinogens in the colon by increasing colonic motility^143^. In addition, coffee has been reported to reduce the synthesis and secretion of bile acids, potential promoters of colon carcinogenesis^144^. The decreased risk of these types of cancers may attribute to some potential mechanism, however, there are many controversies about the mechanisms. Although a positive association was observed between coffee intake and lung cancer, it has been reported that high intakes of coffee are frequently associated with cigarette smoking^145^, which could contribute to the increased risk of lung cancer. However, we take a conservative responsibility for the results because of the limited study numbers and the absence of analysis between smokers and nonsmokers.

Our study demonstrates that coffee intake can reduce the risk of oral, pharynx cancer, colon cancer, liver cancer, prostate cancer endometrial cancer and melanoma by 31%, 13%, 54%, 11%, 27% and 11% respectively for the highest versus lowest coffee intake. Furthermore, coffee intake could reduce the risk of liver cancer, prostate cancer and endometrial cancer by 27%, 3% and 12% with an increment of 2 cups of coffee intake. Inversely, coffee intake seems could increase the risk of lung cancer by 118%. However, considering the shortcomings of our research, our conclusions should be carefully considered. Further studies are needed to clarify the potential underlying mechanisms by which coffee intake may reduce cancer risk. Perhaps add that further studies could assess the association among never smokers, particularly for smoking-related lung cancer.

## Additional Information

**How to cite this article**: Wang, A. *et al*. Coffee and cancer risk: A meta-analysis of prospective observational studies. *Sci. Rep.* **6**, 33711; doi: 10.1038/srep33711 (2016).

## Supplementary Material

Supplementary Information
