# Supplementary material for: Coffee and cancer risk: A meta-analysis of prospective observational studies
Source: Sci Rep. 2016 Sep 26;6:33711. doi: 10.1038/srep33711 (PMC5036059; doi:10.1038/srep33711)
Supplement: Supplementary Information [file srep33711-s1.doc]

Coffee and cancer risk: A meta-analysis of prospective observational studies

Anqiang Wang^1＃^, Shanshan Wang^1＃^, Chengpei Zhu^1＃^, Hanchun Huang^1^, Liangcai Wu^1^, Xueshuai Wan^1^, Xiaobo Yang^1^, Haohai Zhang^1^, Ruoyu Miao^2^, Lian He^1^, Xinting Sang^1＊^, Haitao Zhao^1＊^

STable1a: Characteristics of prospective studies on digestive system cancers in meta-analysis

STable2a: Characteristics of prospective studies on urinary system cancers in meta-analysis

STable3a: Characteristics of prospective studies on female genital system cancers in meta-analysis

STable4a: Characteristics of prospective studies on other cancer in meta-analysis

STable1b: Subgroup analyses of coffee intake and risk of digestive system cancers, meta-regression analysis

STable2b: Subgroup analyses of coffee intake and risk of urinary system cancers, meta-regression analysis

STable3b: Subgroup analyses of coffee intake and risk of female genital system cancers, meta-regression analysis

STable4b: Subgroup analyses of coffee intake and risk of other cancer

**STable1a: Characteristics of prospective studies on digestive system cancers in meta-analysis**

|  | | | | | | | | | | |
| --- | --- | --- | --- | --- | --- | --- | --- | --- | --- | --- |
| **Study/Years**  **of**  **Publication** | Country | No. Cases/Participants | | Subtype of cancer | Duration of follow-up (year) | | Adjusted Factors | Comparison of Exposure Level | Adjusted OR/RR (95% CI) | P value |
| **Jacobsen.**  **1986** | Norway | 38/16555 | | Buccal cavity and pharynx | | 11.5 | age at start of follow-up, for sex, and for region and urban or rural place of residence. " | ≥7cups/day VS ≤2cups/day | 0.90 (0.23, 3.50) | 0.884 |
| **Stensvold. 1994** | Norway | 33/42973 | | Buccal cavity pharynx | | 10.1 | age, cigarettes per day and county of residence as covariates | ≥7cups/day VS <2cups/day | 0.65 (0.25, 1.71) | 0.38 |
| **Naganuma. 2008** | Japan | 48/38679 | | Oral cavity/pharynx | | 12.8 | Age in years, sex, body mass index, alcohol consumption, cigarette smoking, consumption of vegetables and fruit, and green tea consumption | ≥1cups/day VS never | 0.35 (0.16, 0.77) | 0.009 |
| **Ren.**  **2010** | USA | 569/481563 | | oral cavity  Pharynx | | 6 | Age, sex, tobacco smoking, alcohol drinking, BMI, education, ethnicity, usual physical activity throughout the day, vigorous physical activity and daily intake of fruit, vegetables, red meat, white meat, and calories | >3cups/day VS <1cup/day | 0.94 (0.73, 1.23) | — |
| **Tverdal. 2011** | Norway | 354/389624 | | Buccal cancer | | 14.4 | Sex, daily smoking, body mass index and education | ≥9cups/day VS <1cups/day | 0.96 (0.68, 1.36) | 0.92 |
| **Janet.**  **2013** | USA | 868/968432 | | Oral/Pharyngeal Cancer | | 26 | age, sex, race, education, body mass index, alcohol use, smoking, vegetable intake, tea consumption | >2cups/day VS  no/occasional | 0.51 (0.41, 0.64) | — |
| **Esophageal cancer** | | | | | | | | | | |
| **Jacobsen.**  **1986** | Norway | 15/16555 | Esophagus cancer | | | 11.5 | age at start of follow-up，sex, and for region and urban or rural place of residence | ≥7cups/day VS ≤2cups/day | 1.58(0.35, 7.08) | 0.548 |
| **Ishikawa.**  **2006** | Japan | 78/26723 | Esophagus cancer | | | 16.6 | Age in years, cigarette，smoking, alcohol drinking, green tea consumption, and  black tea consumption" | ≥3cups/day VS never | 0.94 (0.36, 2.45) | — |
| **Naganuma.**  **2008** | Japan | 112/38701 | Esophagus cancer | | | 11 | Age in years, sex, body mass index, alcohol consumption, cigarette smoking, consumption  of vegetables and fruit, and green tea consumption | ≥1cups/day VS never | 0.60 (0.37, 0.97) | 0.05 |
| **Ren.**  **2010** | United States | 428/481563 | Squamous Cell Carcinoma Adenocarcinoma | | | 6 | Age, sex, tobacco smoking, alcohol drinking, BMI, education, ethnicity, usual physical activity throughout the day, vigorous physical activity and daily intake of fruit, vegetables, red meat, white meat, and calories | >3cups/day VS <1cup/day | 0.95 (0.70, 1.29) | — |
| **Tverdal.**  **2011** | Norway | 96/389624 | Squamous oesophageal cancer | | | 14.4 | Sex, daily smoking, body mass index and education | ≥9cups/day VS <1cup/day | 0.97 (0.50, 1.88) | 0.67 |
| **Zamora-Ros.2014** | European | 339/442143 | Squamous Cell Carcinoma Adenocarcinoma | | | 11.1 | educational level, smoking status and intensity, physical activity, energy intake, and daily consumption of fruit, vegetables, red and processed meat and coffee and tea mutually, BMI, alcohol intake at baseline. | >477ml/day VS <150ml/day | 0.84 (0.59, 1.20) | 0.715 |
| **Stomach cancer** | | | | | | | | | | |
| **Jacobsen.**  **1986** | Norway | 147/16555 | | Stomach cancer | | 11.5 | "age at start of follow-up (10-yr  intervals), for sex, and for region and urban or rural place of residence. " | ≥7cups/day VS ≤2cups/day | 1.32 (0.75, 2.32) | 0.33 |
| **Nomura.**  **1986** | USA | 106/7355 | | Stomach cancer | | __ | "Age, year of smoking, smoking  status at exam, past smoking  status, number of cigarettes  smoked per day" | ≥5cups/day VS never | 1.18 (0.61, 2.27) | 0.62 |
| **Stensvold. 1994** | Norway | 78/42973 | | Stomach cancer | | 10.1 | age, cigarettes per day and county of residence as covariates. | ≥7cups/day VS <2cups/day | 0.69 (0.34, 1.39) | 0.296 |
| **Galanis.**  **1998** | USA | 108/11799 | | Stomach cancer | | 14.8 | "Age, sex, smoking (M),  education and place of  birth." | ≥7cups/day VS never | 1.80 (0.99, 3.27) | — |
| **Van.**  **1998** | Netherlands | 146/58279 | | Stomach cancer | | 4.3 | — | >4cups/day VS 3cups/day | 1.43 (0.93, 2.19) | — |
| **Tsubono. 2001** | Japan | 419/26311 | | Stomach cancer | | 9 | Age, sex, smoking,  consumption of tea,  alcohol, rice, meat,  vegetables, fruits and beanpast soup, and type of  health insurance | ≥4cups/day VS never | 1.00 (0.61, 1.63) | — |
| **Larsson.**  **2006** | Sweden | 160/61433 | | Stomach cancer | | 15.7 | "Age, BMI, smoking, PA, family history of CRC  and diabetes, aspirin use, multivitamin use,  energy, red meat, fruits, vegetables, milk,  for women postmenopausal hormone use" | ≥4cups/day VS  1 cup/day | 1.86 (1.24, 2.78) | — |
| **Nilsson.**  **2010** | Sweden | 70/61569 | | Stomach cancer | | 6 | age, sex, BMI, smoking, education, and recreational physical activity | ≥4cups/day VS <1cups/day | 0.99 (0.44, 2.22) | 0.168 |
| **Ren.**  **2010** | United States | 454/418563 | | Gastric cardia Gastric non-cardia | | 6 | Age, sex, tobacco smoking, alcohol drinking, BMI, education, ethnicity, usual physical activity throughout the day, vigorous physical activity and daily intake of fruit, vegetables, red meat,  white meat, and calories | >3cups/day VS <1cups/day | 1.30 (0.96, 1.76) | — |
| **Bidel.**  **2013** | Finland | 299/60041 | | Stomach cancer | | 18 | Age, sex, BMI, study year,  education, smoking,  physical activity, history of  diabetes, and consumption  of tea and alcohol. sex | ≥10cups/day VS never | 0.75 (0.40, 1.41) | 0.19 |
| **Cheryl.**  **2014** | Singapore | 647/63257 | | Stomach cancer | | 14.1 | "Age, BMI, gender,  interview year, dialect,  education, smoking,  total energy intake | Daily VS never | 0.85 (0.69, 1.04) | — |
| **Sanikini. 2015** | European | 683/477312 | | Stomach cancer | | 13.8 | smoking, BMI, physical activity, education level, diabetes, alcohol consumption, intake of energy, fiber, vegetable, fruit, fish and red and processed meat." | Q4 VS Q1 | 1.09 (0.84, 1.43) | — |
| **Colorectal cancer** | | | | | | | | | | |
| **Jacobsen.**  **1986** | Norway | 163/16555 | | Rectum and colon | | 11.5 | age at start of follow-up, for sex, and for region and urban or rural place of residence | ≥7cups/day VS ≤2cups/day | 0.60 (0.31, 1.14) | 0.117 |
| **Nomura.**  **1986** | USA | 168/7355 | | Rectum and colon | | __ | age at examination, years of smoking, number of cigarettes smoked per day, current smoking status at examination, and past smoking status | ≥5cups/day VS never | 0.86 (0.37, 2.01) | 0.773 |
| **Wu.**  **1987** | USA | 126/11644 | | colorectal | | 4.5 | Age | ≥4cups/day VS <1cup/day | 1.36 (0.69, 2.69) | 0.371 |
| **Klatsky. 1988** | USA | 269/106203 | | Rectum and colon | | __ | Age, sex, alcohol, smoking, BMI, race, education, serum cholesterol | Yes VS no | 0.90 (0.80, 1.02) | 0.086 |
| **Stensvold. 1994** | Norway | 209/42973 | | Rectum and colon | | 10.1 | age, cigarettes per day and county of residence as covariates. | ≥7cups/day VS <2cups/day | 0.87 (0.53, 1.43) | — |
| **Hartman.**  **1998** | Finland | 285/27111 | | Rectum and colon | | 6.1 | age, intervention group, calcium, occupational physical activity, BMI, and tea | >6cups/day VS <4cups/day | 0.80 (0.54, 1.18) | 0.259 |
| **Terry.**  **2001** | Sweden | 460/61463 | | Rectum and colon | | 9.6 | age，body mass index, education level), quartiles of intakes of energy and red meat, and quartiles of energy adjusted total fat, dietary fiber, calcium, vitamin C, folic acid, vitamin D, and alcohol | ≥4cups/day VS <1cups/day | 1.04 (0.70, 1.54) | 0.95 |
| **Michels.**  **2005** | USA | 1431/173229 | | Rectum and colon | | __ | Age, BMI, height, alcohol, smoking, PA, family history of CRC, aspirin use,  vitamin supplement, energy, red meat | >5cups/day VS never | 0.98 (0.69, 1.39) | 0.60 |
| **Mucci.**  **2006** | Sweden | 741/61467 | | colorectal cancer | | __ | Age at screening, BMI, education, alcohol, energy, saturated fat, fiber | ≥4cups/day VS ≤1cups/day | 1.00 (0.73, 1.36) | — |
| **Larsson.**  **2006** | Sweden | 723/81922 | | Colorectal cancer | | __ | Age, BMI, smoking, PA, family history of CRC and diabetes, aspirin use, multivitamin use, energy, red meat, fruits, vegetables, milk, for women postmenopausal hormone use | ≥6cups/day VS <1cups/day | 1.06 (0.74, 1.52) | — |
| **Oba.**  **2006** | Japan | 213/30221 | | colon cancer | | __ | age, height, BMI, total pack-years of cigarette smoking, alcohol intake, physical activity, black tea intake and green tea/coffee intake." | >1cup/day VS <1cup/month | 0.63 (0.41, 0.96) | 0.033 |
| **Naganuma.**  **2007** | Japan | 457 /38701  " | | colorectal cancer | | 11 | Age, sex, BMI, alcohol, smoking, walking time, family history, education, energy, fruits, vegetables, meat, tea; for F, menopausal status, numbers of pregnancies and deliveries, age at menarche, age at first delivery | ≥3cups/day VS never | 0.95 (0.65, 1.39) | 0.55 |
| **Lee.**  **2007** | Japan | 1163/96162 | | colorectal cancer | | 10 | BMI, smoking status, alcohol drinking, family history of colorectal cancer, physical activity, and intake of green  vegetables, beef, pork, green tea, Chinese tea and black tea | ≥3cups/day VS almost never | 0.91 (0.57, 1.44) | 0.895 |
| **Peterson.**  **2010** | Singapore | 961/61321 | | Rectum and colon | | 9.8 | age at baseline, gender, dialect group, year of recruitment, level of education, body mass index, cigarette smoking, alcohol consumption, physical activity, history of diabetes, family history of colorectal cancer, and green tea intake. | ≥2cups/day VS <1cups/day | 0.98 (0.76, 1.27) | 0.895 |
| **Bidel.**  **2010** | Finland | 538/60041 | | colorectal cancer | | 18 | Age, sex, study year, education, cigarette smoking, alcohol consumption, leisure-time PA, history of diabetes, tea consumption, BMI | ≥10cups/day VS 01cups/day | 1.03 (0.58, 1.83) | 0.61 |
| **Nilsson.**  **2010** | Sweden | 321/61569 | | Colorectum cancer | | 6 | age, sex, BMI, smoking, education, and recreational physical activity | ≥4occasions/day VS<1occasion/day | 1.43 (0.86, 2.38) | 0.168 |
| **Simons.**  **2010** | Holland | 2199/120852 | | Colorectum | | 13.3 | Age, family history of CRC, smoking, educational, BMI, ethanol, meat, processed meat, folate, vitamin B6, fibre, fluid | >6cups/day VS ≤2cups/day | 1.03 (0.81, 1.30) | 0.82 |
| **Rashmi.**  **2012** | USA | 6946/489706 | | Colorectal | | 10.5 | age, sex, race, education, smoking status, time since quitting for former smokers, smoking dose, ever smoke a pipe or cigar, diabetes, colorectal screening, family history of colorectal cancer, regular nonsteroidal antiinflammatory drug use, marital status, BMI, frequency of vigorous physical activity, calories, fruit and vegetables, red meat, dietary calcium intake, alcohol, and menopausal hormone therapy in women. | ≥6cups/day VS none | 0.80 (0.69, 0.93) | 0.008 |
| **Dominianni.**  **2013** | USA | 681/57398 | | colorectal | | 11.4 | age, gender, race, family history of colorectal cancer, education, body mass index, physical activity, smoking status, NSAID intake, history of  diabetes, number of colorectal examinations up to 3 years before the start of study, hormone use, fruit intake, vegetable intake, meat intake, alcohol intake and study centre. | ≥4cups/day VS none | 1.08 (0.79, 1.48) | 0.229 |
| **Hiroya.**  **2014** | Japan | 1001/ 58221 | | colorectal cancer | | __ | age, smoking, drinking, family history of colorectal cancer, education, body mass index, walking time, and regular  meat consumption, and distict." | ≥4cups/day VS <1cup/day | 1.54 (1.00, 2.35) | 0.049 |
| **Vincent.**  **2014** | European | 4234/477071 | | colorectal cancer | | 11.6 | body mass index, diabetes mellitus, menopausal status, hormone replacement therapy, physical activity, educational level, smoking status, and baseline intake of energy from fat, energy from non-fat, alcohol, fibers, dairy products, red meat and processed meat | Q5 VS Q1 | 1.06 (0.95, 1.18) | 0.58 |
| **Pancreatic cancer** | | | | | | | | | | |
| **Nomura.**  **1981** | Japan | 28/8004 | | pancreatic cancer | | 13 | age and smoking status  (never, past, current cigarette smoker). | >5cups/day VS never | 2.91(0.63, 13.50) | 0.085 |
| **Whittemore.**  **1983** | USA | 126/50000 | | pancreatic cancer | | __ | age, college, and class year | 》2cups/day VS <2cups/day | 1.10 (0.67, 1.81) | — |
| **Jacobsen.**  **1986** | Norway | 39/16555 | | pancreatic cancer | | 11.5 | age, sex and residence | 》7cups/day VS 《2cups/day | 1.17 (0.39, 3.50) | 0.94 |
| **Nomura.**  **1986** | America | 21/7355 | | pancreatic cancer | | __ | "adjusted for age at examination, years of smoking (whether past or current), number  of cigarettes smoked per day (whether past or current), smoking  status at exam (no/yes), and past smoking status (no/yes)." | 》5cups/day VS never | 1.90 (0.38, 9.45) | 0.411 |
| **Hiatt.**  **1988** | Northern California | 49/122894 | | pancreatic cancer | | 6 | — | >4cups/day VS never | 0.70 (0.23, 2.16) | — |
| **Shibata.**  **1994** | Canada | 63/13979 | | pancreatic cancer | | 9 | "Adjusted for sex  and age" | 》4cups/day VS <1cups/day | 0.88 (0.28, 2.78) | — |
| **Stensvold.**  **1994** | Norway | 41/42973 | | pancreatic cancer | | 10.1 | "With age, cigarettes per day  and county of residence as covariates" | 》7cups/day VS <2cups/day | 2.06 (0.52, 8.12) | 0.303 |
| **Lisa.**  **1997** | USA | 66/33976 | | pancreatic cancer | | 9 | "Adjusted for age, smoking status,  and pack-years of smoking." | 》17.5cups/week VS <7cups/week | 2.15 (1.08, 4.29) | 0.03 |
| **Michaud.**  **2001** | United States | 288/173229 | | pancreatic cancer | | __ | "adjusted for age in 5-year categories, pack-years of smoking (past 15 years; current and past smokers separately), BMI (quintiles at baseline),  history of diabetes mellitus, history of cholecysectomy, energy intake (quintiles), and period" | >cups/day VS never | 0.62 (0.27, 1.43) | 0.35 |
| **Isaksson.**  **2002** | Swedish | 131/21884 | | pancreatic cancer | | 16 | sex, age and cigarette  smoking | 》7cups/day VS never | 0.39 (0.17, 0.89) |  |
| **Stolzenberg-Solomon.2002** | Finland | 163/27111 | | pancreatic cancer | | 10.2 | age and years of smoking | Q5 VS Q1 | 0.95 (0.54, 1.68) | 0.62 |
| **Juhua.**  **2007** | Japan | 233/102137 | | pancreatic cancer | | 11 | "body mass index, leisure-time physical  activity in terms of frequency of sports, smoking status, alcohol intake, history of diabetes, history of cholelithiasis, study area, and age | 》3cups/day VS rarely | 0.80 (0.44, 1.44) | 0.4 |
| **Nilsson.**  **2010** | Sweden | 74/61569 | | pancreatic cancer | | 6 | age, sex, BMI, smoking, education, and recreational physical activity | ≥4cups/day VS <1cups/day | 1.50 (0.57, 3.92) | 0.412 |
| **Siamak.**  **2013** | Finland | 235/60041 | | pancreatic cancer | | 18 | Age, year, education, smoking, alcohol consumption, leisure time physical activity, history of diabetes, tea consumption, and body mass index. | 》10cups/day VS never | 0.82 (0.38, 1.76) | 0.95 |
| **Nirmala.**  **2013** | European | 865/477312 | | pancreatic cancer | | 11.6 | sex, center, and age at diagnosis in 1-year categories, and adjusted for height, weight, smoking status, history of diabetes, highest  attained education, and physical activity | Q5 VS Q1 | 1.03 (0.83, 1.27) | 0.67 |
|  |  |  | |  | |  | **Liver cancer** |  |  |  |
| **Shimazu.**  **2005** | Japan | 117/61107 | | Primary liver cancer | | __ | Age, sex, smoking, alcohol drinking, history of liver disease | ≥2cups/d VS never | 0.58 (0.36, 0.96) | 0.024 |
| **Inoue.**  **2005** | Japan | 334/90452 | | HCC | | 10 | Age, sex, study area,  smoking, and  intakes of alcohol,  green tea, and  green vegetables | >5cups/d VS never | 0.24 (0.08, 0.74) | <.001 |
| **Hu.2008** | Finland | 128/60323 | | HCC | | 19.3 | Age, sex, smoking, alcohol drinking, education, study year, diabetes and chronic liver disease, BMI and during follow up. | ≥8cups/d VS 0-1cups/d | 0.32 (0.16, 0.63) | 0.003 |
| **Ohishi.**  **2008** | Japan | 224/644 | | HCC | | __ | Hepatitis virus infection, alcohol consumption, smoking habits, BMI, diabetes mellitus, and radiation dose to the liver | Daily VS never | 0.40 (0.16, 1.01) | 0.055 |
| **Inoue.**  **2009** | Japan | 110/18815 | | HCC | | 12.7 | "Age, sex, area, smoking, alcohol drinking, BMI,  diabetes mellitus, green tea consumption,  serum ALT level, and HBV and HCV infection  status" | ≥3cups/d VS never | 0.54 (0.21, 1.39) | 0.025 |
| **Johnson.**  **2011** | Chinese | 362/61321 | | HCC | | __ | "Age at recruitment, sex, dialect group,  year of recruitment, BMI, level of education,  consumption of alcoholic beverages,  smoking, black tea and green tea intake,  and history of diabetes" | ≥3cups/d VS never | 0.56 (0.31, 1.01) | 0.05 |
| **Lai.**  **2013** | Finland | 194/27034 | | HCC | | 18.2 | ATBC intervention arm (categorical), age (continuous), BMI (continuous), education (elementary school education or less, higher than elementary school education), marital status  (currently married, not), history of diabetes (yes, no), years of smoking (continuous), cigarettes smoked per day (continuous), alcohol (continuous), tea intake (non-drinkers, drink up to 6 oz, drink  46 oz) and serum cholesterol (continuous) | ≥4cups/d VS 0-1cups/d | 0.53 (0.30, 0.95) | 0.0007 |
| **Setiawan.**  **2015** | US | 451/162022 | | HCC | | 18 | age, sex, and race/ethnicity. education, marital status, history of diabetes, years of smoking, cigarettes smoked per day, alcohol, tea intake  and serum cholesterol | >4cups/d VS never | 0.59 (0.35, 0.99) | 0.0002 |
| **Bamia1.**  **2015** | European | 201/486799 | | HCC | | 11 | sex, diabetes mellitus (self-reported at enrolment), education, body mass index, smoking, physical activity, alcohol intake | Q5 VS Q1 | 0.28 (0.16, 0.49) | <0.001 |

**Stable2a: Characteristics of prospective studies on urinary system cancer in meta**-analysis

| **Renal cancer** | | | | | | | | |  |
| --- | --- | --- | --- | --- | --- | --- | --- | --- | --- |
| **Study/Years**  **of**  **Publication** | Country | No. Cases/Participants | Subtype of cancer | Duration of follow-up (year) | Adjusted Factors | Comparison of Exposure Level | Adjusted OR/RR (95% CI) | P value |  |
| **Jacobsen.**  **1986** | Norway | 44/16555 | renal cancer | 11.5 | age at start of follow-up, for sex, and for region and urban or rural place of residence | ≥7cups/day VS ≤2cups/day | 0.30 (0.07, 1.33) | 0.112 |  |
| **Stensvold. 1994** | Norway | 43/42973 | renal cancer | 10.1 | age, cigarettes per day and county of residence as covariates. | ≥7cups/day VS <2cups/day | 1.01 (0.38, 2.67) | 0.985 |  |
| **Lee.**  **2005** | USA | 246/136587 | renal cancer | 14 | In the NHS, multivariate RRs were adjusted for BMI, history of hypertension, parity, history of diabetes, smoking status, and total energy intake. In the HPFS, multivariate RRs were adjusted for BMI, history of hypertension, smoking status, multivitamin use, and total energy intake. Models of all beverages, except alcoholic beverages, were additionally adjusted for alcohol intake (continuous).  Additionally adjusted for two other alcoholic beverages (continuous). | 1-3cups/day VS <1cup/month | 0.87 (0.62, 1.22) | — |  |
| **Allen. 2011** | UK | 588/778781 | renal cell carcinoma | 5.2 | socioeconomic status, body mass index, and smoking. | ≥12drinkings/day VS 1-7drinkings/day | 1.05 (0.78, 1.42) | 0.4 |  |
| **Nilsson.**  **2010** | Sweden | 56/61569 | renal cell cancer | 6 | age, sex, BMI, smoking, education, recreational physical activity | ≥4occasions/day VS <1occasions/day | 0.30 (0.11, 0.80) | 0.014 |  |
| **Bladder cancer** | | | | | | | | |  |
| **Jacobsen.**  **1986** | Norway | 94/16555 | Bladder cancer | 11.5 | age at start of follow-up, sex, and for region and urban or rural place of residence. " | ≥7cups/day VS ≤2cups/day | 0.98 (0.51, 1.89) | — |  |
| **Nomura.**  **1986** | USA | 42/7355 | Bladder cancer | 10 | age at examination, years of smoking, number of cigarettes smoked per day, current smoking status at examination, and past smoking status | ≥5cups/day VS never | 2.76(0.96,7.95) | — |  |
| **Mills.**  **1991** | USA | 52/34198 | Bladder cancer | 6 | Age, sex, and smoking | 2cups/day VS never | 1.99 (0.90, 4.39) | — |  |
| **Chyou.**  **1993** | USA | 96/7992 | Bladder cancer | 22 | Age and smoking | ≥5cups/day VS <1cups/day | 2.07 (0.84, 5.11) | 0.174 |  |
| **Stensvold.**  **1994** | Norway | 53/42973 | Bladder cancer | 10.1 | age, cigarettes per day and county of residence as covariates. | ≥7cups/day VS ≤2cups/day | 2.76 (0.96, 7.95) | — |  |
| **Michaud.**  **1999** | USA | 252/47909 | Bladder cancer | 10 | Geographic region, age, pack-years of smoking, current smoking status, energy intake, and intake of fruits and vegetables | ≥4cups/day VS <1cups/month | 0.79 (0.48, 1.30) | 0.56 |  |
| **Nagano.**  **2000** | Japan | 89/38540 | Bladder cancer | 13 | Age, gender, radiation dose, smoking status, education level, body-mass index, and calendar time | ≥5cups/week VS 0 | 0.90 (0.52, 1.56) | 0.78 |  |
| **Zeegers.**  **2001** | Netherlands | 569/3123 | Bladder cancer | 6.3 | age, number of cigarettes/day, years of cigarette smoking, and tea consumption | ≥7cups/week VS 4-5cups/day | 0.95 (0.69, 1.30) | — |  |
| **Apeksha.**  **2002** | USA | 110/37459 | Bladder cancer | 13 | Age | ≥4cups/day VS never or 1cup/month | 1.59 (0.95, 2.67) | — |  |
| **Kurahashi.**  **2009** | Japan | 206/104440 | Bladder cancer | 12.6 | Age, area, smoking status, alcohol drinking and green tea" | >3cups/day VS 0 | 1.02 (0.62, 1.68) | — |  |
| **Prostate cancer** | | | | | | | | |  |
| **Jacobsen.**  **1986** | Norway | 260/16555 | prostate cancer | 11.5 | age at start of follow-up, for sex, and for region and urban or rural place of residence | ≥7cups/day VS ≤2cups/day | 0.66 (0.36, 1.21) | 0.076 |  |
| **Nomura.**  **1986** | USA | 120/7355 | Prostate cancer | 10 | age at examination, years of smoking, number of cigarettes smoked per day, current smoking status at examination, and past smoking status | ≥5cups/day VS never | 1.43 (0.79, 2.60) | 0.233 |  |
| **Severson.**  **1989** | USA | 174/7999 | Prostate Cancer | __ | Age | ≥5cups/day VS ≤1cups/day | 0.92 (0.59, 1.44) | — |  |
| **Le.**  **1994** | USA | 198/20316 | Prostate Cancer | __ | Age, ethnicity, and income | Q4 VS Q1 | 1.10 (0.71, 1.71) | 0.43 |  |
| **Stensvold. 1994** | Norway | 38/42973 | Prostate Cancer | 10.1 | age, cigarettes per day and county of residence as covariates. | ≥7cups/day VS <2cups/day | 0.60 (0.24, 1.48) | 0.267 |  |
| **Ellison.**  **2000** | Canada | 145/39750 | Prostate Cancer | __ | age and wine consumption | >750ml/day VS <250ml/day | 1.42 (0.77, 2.61) | — |  |
| **Allen.**  **2004** | Japan | 196/18115 | Prostate Cancer | 16.9 | age, calendar period, city of  residence, radiation dose and education level" | Almost daily VS <2times/week | 1.02 (0.71, 1.46) | 0.88 |  |
| **Nilsson.**  **2010** | Sweden | 653/30930 | Prostate Cancer | 6 | age, sex, BMI, smoking, education, and recreational physical activity | ≥4cups/day VS <1cups/day | 1.03 (0.77, 1.38) | 0.834 |  |
| **Wilson.**  **2011** | USA | 5035/97911 | Prostate Cancer | 20 | Race, height, BMI at age 21, current BMI, vigorous physical activity, smoking, diabetes, family history of prostate cancer in father or brother, multivitamin use, intakes of processed meat, tomato  sauce, calcium, a-linolenic acid,  supplemental vitamin E, alcohol intake, energy intake and history of PSA testing | ≥6cups/day VS none | 0.82 (0.68–0.98) | — |  |
| **Shafique.**  **2012** | UK | 318/6017 | Prostate Cancer | 28 | Age at screening, cholesterol, systolic blood pressure, BMI, alcohol intake, tea consumption, smoking status, social class | ≥3cups/day VS 0 | 0.93 (0.21, 4.18) | — |  |
| **Bosire.**  **2013** | USA | 23335/288391 | Prostate Cancer | 10.5 | Age, race, height, BMI, physical activity, history of diabetes, family history of prostate cancer, PSA testing, intakes of tomato sauce, alpha-linolenic acid, and total energy intake | ≥6cups/day VS none | 0.94 (0.87, 1.02) | 0.08 |  |
| **Li.**  **2013** | Japan | 318/18853 | Prostate Cancer | 11 | Age, education level, BMI, time engaging in sports or exercise, marital status, time spent walking,  smoking status, family history of cancer, consumption of tea | ≥3cups/day VS never | 0.63 (0.39, 1.01) | 0.02 |  |
| **Discacciati. 2013** | Sweden | 3286/44613 | Prostate Cancer | 13 | age, smoking, BMI, height, physical activity, total cholesterol, triglycerides, systolic blood pressure, year of examination and diabetes. | ≥6cups/day VS none | 0.82 (0.72, 0.95) | — |  |
| **Tverdal.**  **2015** | Norway | 3286/224234 | Prostate Cancer | __ | Tea, alcohol, BMI, personal history of diabetes, family history of prostate cancer, family history  of prostate cancer, physical activity, education, total energy intake | ≥9cups/day VS none | 0.78 (0.69, 0.89) | — |  |

**Stable3a: Characteristics of prospective studies on female genital system cancers in meta**-analysis

| **Breast cancer** | | | | | | | | |
| --- | --- | --- | --- | --- | --- | --- | --- | --- |
| **Study/Years**  **of**  **Publication** | Country | No. Cases/Participants | Subtype of cancer | Duration of follow-up (year) | Adjusted Factors | Comparison of Exposure Level | Adjusted OR/RR (95% CI) | P value |
| **Jacobsen.**  **1986** | Norway | 470/16555 | Breast Cancer | 11.5 | Adjusted for age, sex and residence | ≥7cups/day VS ≤2cups/day | 0.55 (0.31, 0.97) | 0.7 |
| **Vatten.**  **1990** | Norway | 152/14593 | breast cancer | 12 | — | ≥7cups/day VS ≤2cups/day | 0.80 (0.48, 1.34) | 0.37 |
| **Høyer.**  **1992** | Denmark | 51/5207 | breast cancer | 26 | — | ≥7cups/day VS <2cups/day | 1.70 (0.69, 4.21) | P > 0.20 |
| **Folsom.**  **1993** | USA | 580/34388 | breast cancer | 5 | "Adjusted for age, walst/hlp ratio, number of livebirths, age at first livebirth, age at menarche, family history of breast cancer,  family history x walst/hlp ratio, and family history x number of livebirttis" | ≥4cups/day VS never or <1cup /month | 1.02 (0.80, 1.31) | — |
| **Stensvold.**  **1994** | Norway | 311/42973 | breast cancer | 10.1 | "With age, cigarettes per day  and county of residence as covariates" | ≥7cups/day VS <2cups/day | 1.22 (0.92, 1.62) | — |
| **Key.**  **1999** | Japan | 344/34759 | breast cancer | __ | adjusted for attained age, calendar period, city, age at time of bombing and radiation dose | ≥5cups/day VS ≤1cups/day | 1.19 (0.93, 1.52) | — |
| **Michels.**  **2002** | Sweden | 1271/59036 | breast cancer | 9.5 | Age , family history of breast cancer, height, body mass index, education, parity, age at first birth，alcohol consumption, total caloric intake | ≥5cups/day VS ≤1cups/week | 0.94 (0.72, 1.23) | — |
| **Suzuki.**  **2004** | Japan | 95/4396 | breast cancer | 6.6 | "Adjusted for age, smoking, number of children, use of oral contraception, family history of breast cancer,  and menopausal status" | ≥253ml/day VS ≤111ml/day | 1.10 (0.66, 1.84) | 0.71 |
| **Ganmaa.**  **2008** | USA | 5272/85987 | breast cancer | 22 | "Adjusted for: age months, smoking status, body mass index, physical activity, height, alcohol intake, family history of breast cancer in mother or a sister, history of benign breast disease, menopausal status, age at menopause, use of hormone therapy, age at menarche, parity and age at first, birth, weight change after 18 and duration of postmenopausal hormone use (continuous). | ≥4cups/day VS <1cups/month | 0.92 (0.82, 1.03) | 0.14 |
| **Ishitani.**  **2008** | USA | 1181/38432 | breast cancer | 10 | "Adjustments for age, randomized treatment assignment, body mass index, physical activity, total energy intake, alcohol intake, multivitamin use, age at  menopause, age at menarche, age at first pregnancy lasting ≥6months, number of pregnancies lasting ≥6months, menopausal status, postmenopausal  hormone use, prior hysterectomy, prior bilateral oophorectomy, smoking status, family history of breast cancer in mother or a sister, and history of benign  breast disease" | ≥4cups/day VS almost never | 1.08 (0.89, 1.31) | 0.27 |
| **Larsson.**  **2009** | Sweden | 2952/ 61433 | breast cancer | 17.4 | age, education (primary school, high school, university), body mass index, height, parity, age at first birth, age at menarche, age at menopause, use of oral contraceptives (ever/never), use of postmenopausal hormones, family history of breast cancer (yes/no), and intakes of total energy, alcohol, and tea | ≥4cups/day VS <1cups/month | 1.02 (0.87, 1.20) | 0.74 |
| **Wilson.**  **2009** | USA | 1179/ 90628 | breast cancer | 14 | Multivariable models are stratified by age in months and calendar year and adjusted for the following: body mass index, height, oral contraceptive use, parity and age at first birth, age at menarche, family history of breast cancer, history of benign breast disease, smoking, physical activity, animal fat, glycemic load, alcohol intake (continuous), and total energy intake | Q5 VS Q1 | 0.92 (0.77, 1.10) | 0.28 |
| **Bhoo.**  **2010** | European | 681/127323 | breast cancer | 9.6 | "Adjusted to propensity score (based on age, smoking status, educational status, BMI, alcohol intake, energy intake, energy-adjusted saturated  fat intake, energy-adjusted fiber intake, tea intake, physical activity level, ever prior use of oral contraceptives, presence of hypercholesterolemia,  family history of breast cancer, age at menarche, parity, and cohort)" | 5cups/day VS never | 0.94 (0.72, 1.23) | — |
| **Bogg.**  **2010** | USA | 1268/52062 | breast cancer | 12 | "Adjusted for age, energy intake, age at menarche, BMI at age 18, family history of breast cancer, education, geographic region, parity, age at first  birth, oral contraceptive use, menopausal status, age at menopause, female hormone use, vigorous activity, smoking status, and alcohol intake" | ≥4cups/day VS <1cups/month | 1.03 (0.77, 1.38) | 0.9 |
| **Nilsson.**  **2010** | Sweden | 588/63603 | breast cancer | 6 | "Multivariate models adjusted for age, sex,  BMI, smoking, education, and recreational physical activity" | ≥4cups/day VS <1cups/day | 0.92 (0.68, 1.25) | 0.603 |
| **Fagherazzi.**  **2011** | French | 2868/67703 | Breast cancer | 11 | total energy intake, ever use of oral contraceptives, age at menarche, age at menopause, number of children, age at first pregnancy, history of breast cancer in the family and years of schooling, current use of  postmenopausal hormone therapy, personal history of benign breast disease, menopausal status and BMI | >3cups/day VS never | 1.02 (0.90, 1.16) | 0.79 |
| **Gierach.**  **2012** | USA | 9915/198404 | breast cancer | 5.2 | "adjusting for age, race/ethnicity, education, BMI, smoking status and dose, alcohol, proportion of total energy from fat, age at first live birth, menopausal  hormone therapy use, history of breast biopsy, and family history of breast cancer | ≥4cups/day VS never | 0.98 (0.89, 1.07) | 0.48 |
| **Ovarian cancer** | | | | | | | | |
| **Stensvold. 1994** | Norway | 93/42973 | ovarian cancer | 10.1 | age, cigarettes per day and county of residence as covariates. | ≥7cups/day VS <2cups/day | 2.16 (0.80, 5.83) | 0.128 |
| **Larsson. 2005** | Sweden | 301/61057 | ovarian cancer | 15.1 | Age, BMI, smoking, family history of CRC and diabetes, aspirin use, multivitamin use, energy, red meat, fruits, vegetables, milk, for women postmenopausal hormone use" | ≥4cups/day VS <1cup/day | 1.04 (0.73, 1.50) | — |
| **Silvera.**  **2007** | Canada | 264/49613 | ovarian cancer | 16.4 | age, smoking history, pack-years of smoking, alcohol intake, education, BMI, parity, participation in vigorous physical activity, menopausal status, oral contraceptive use, energy intake, lactose intake, study center, and randomization  group | ≥4cups/day VS 0 | 1.62 (0.95, 2.76) | — |
| **Steevens. 2007** | Netherlans | 280/62573 | ovarian cancer | 13.3 | age , use of oral contraceptives, parity, cigarette smoking, tea | ≥3cups/day VS none | 1.08 (0.75, 1.56) | 0.35 |
| **Tworoger. 2008** | United States | 507/80253 | Epithelial Ovarian Cancer | __ | age, parity, oral contraceptive use, postmenopausal hormone use, tubal ligation, smoking status, and body mass index. | ≥3cups/day VS none | 0.77 (0.59, 1.02) | — |
| **Lueth.**  **2008** | United States | 266/29060 | Epithelial Ovarian Cancer | __ | " age, smoking, BMI, age at menopause, parity, oral contraceptive use, education level, physical activity, and total  energy intake as covariates." | ≥5cups/day VS 0 | 1.28 (0.76, 2.16) | 0.15 |
| **Nilsson.**  **2010** | Sweden | 71/30639 | ovarian cancer | 6 | age, sex, BMI, smoking, education, and recreational physical activity | ≥4occasions/day VS<1occasion/day | 1.41 (0.53, 3.74) | — |
| **Braem. 2012** | European | 1244/330849 | epithelial ovarian  cancer | 11.7 | center and age and adjusted for parity, oral contraceptive use, BMI, smoking status, alcohol consumption， total energy intake, duration of breastfeeding, menopausal status, height, and educational level | Q5 VS Q1 | 1.05 (0.75, 1.46) | — |
| **Endometrial cancer** | | | | | | | | |
| **Jacobsen BK.1986** | Norway | 11/16555 | Corpus cancer | 11.5 | age at start of follow-up, for sex, and for region and urban or rural place of residence | ≥7cups/day VS ≤2cups/day | 0.35 (0.04, 2.93) | 0.333 |
| **Stensvold. 1994** | Norway | 84/42973 | Corpus uteri cancer | 10.1 | age, cigarettes per day and county of residence as covariates | ≥7cups/day VS <2cups/day | 0.80 (0.34, 1.90) | 0.61 |
| **Shimazu. 2008** | Japan | 117/53724 | endometrial cancer | 15 | Age, study center, body mass index,  menopausal status, age at menopause,  use of exogenous hormones, smoking  status, green vegetable consumption,  beef consumption, pork consumption,  green tea consumption | ≥3cups/day VS ≤2days/week | 0.38 (0.16, 0.91) | 0.007 |
| **Friberg. 2009** | Sweden | 677/60634 | endometrial cancer | 17.6 | Age, BMI, smoking, education,  age at menopause, age at  menarche, OC use, PMH use,  parity, history of diabetes, tea,  buns, cookies, cakes, total  energy intake | ≥4cups/day VS ≤1cups/day | 0.75 (0.57, 0.98) | — |
| **Nilsson.**  **2010** | Sweden | 108/30639 | endometrial cancer | 6 | age, sex, BMI, smoking, education, and recreational physical activity | ≥4occasions/day VS<1occasion/day | 0.88 (0.44, 1.77) | 0.719 |
| **Je.**  **2011** | USA | 672/67470 | endometrial cancer | 26 | Age, BMI, age at menarche, age  at menopause, parity and age at  last birth, duration of OC use,  PMH use, alcohol, pack years of  smoking, total energy intake | ≥4cups/day VS <1cups/day | 0.75 (0.57, 0.98) | 0.02 |
| **Giri.**  **2011** | USA | 427/45696 | endometrial cancer | 7.5 | age, ethnicity, unopposed estrogen use, progestin + estrogen us, smoking and BMI | ≥4cups/day VS <1cups/day | 0.86 (0.63, 1.18) | 0.41 |
| **Gunter.**  **2012** | USA | 1486/111429 | endometrial cancer | 9.3 | Age, smoking, BMI, age at  menarche, age at first child’s  birth, parity, age at menopause,  HRT use, diabetes,  physical activity | >3cups/day VS 0 | 0.64 (0.51, 0.80) | 0.004 |
| **Uccella.**  **2013** | USA | 542/23356 | endometrial cancer | 20 | age, diabetes, duration of HT use, hypertension, age at menarche, age at menopause, quartiles of body mass index, waist-to-hip ratio, smoking status, pack years of smoking, total energy and alcohol use | ≥4cups/day VS never | 0.72 (0.53, 0.99) | — |
| **Elisabete.**  **2014** | Sweden | 144/42270 | endometrial cancer | 6 | age, education, duration of hormonal contraceptive use, parity, duration of breastfeeding, smoking status and number of cigarettes/day, menopausal status, body mass index, and diabetes | >3cups/day VS <2cups/day | 0.64 (0.39, 1.06) | 0.1742 |
| **Oxana.**  **2014** | Norway | 462/97926 | endometrial cancer | 10.9 | parity, smoking status, BMI, duration of Oral contraceptives, Hormone replacement therapy | ≥8cups/day VS ≤1cups/day | 0.52 (0.34, 0.79) | 0.003 |
| **Merritt**  **2015** | Europe | 1303/521330 | endometrial cancer | 3.6 | Age, BMI, total energy intake, smoking status, age at menarche, oral  contraceptive use, menopausal status,  postmenopausal hormone use, parity,  the age of recruitment and the study center | 750.0 g/day vs.8.6  g/day | 0.81(0.68,0.97) | — |

**Stable4a: Characteristics of prospective studies on other system cancers in meta**-analysis

| **Lung cancer** | | | | | | | | |
| --- | --- | --- | --- | --- | --- | --- | --- | --- |
| **Study/Years**  **of**  **Publication** | Country | No. Cases/Participants | Subtype of cancer | Duration of follow-up (year) | Adjusted Factors | Comparison of Exposure Level | Adjusted OR/RR (95% CI) | P value |
| **Jacobsen. 1986** | Norway | 177/16555 | Lung cancer | 11.5 | "age at start of follow-up (10-yr  intervals), for sex, and for region and urban or rural place of residence. " | ≥7cups/day VS ≤2cups/day | 1.76 (1.07, 2.89) | 0.025 |
| **Nomura. 1986** | USA | 110/7355 | Lung cancer | 10 | "Age, year of smoking, smoking  status at exam, past smoking  status, number of cigarettes  smoked per day" | ≥5cups/day VS never | 2.33 (1.25, 4.32) | 0.007 |
| **Stensvold. 1994** | Norway | 125/42973 | Lung cancer | 10.1 | age, cigarettes per day and county of residence as covariates. | ≥7cups/day VS <2cups/day | 6.01 (2.42, 14.93) | 0 |
| **Takezaki. 2003** | Japan | 51/5885 | Lung cancer | 14.67 | age (continuous), sex, smoking (never, former, and current), and occupation. | Every day VS never | 1.20 (0.60, 2.40) | 0.56 |
| **Melanoma** | | | | | | | | |
| **Jacobsen.**  **1986** | Norway | 19/16555 | Melanoma | 11.5 | age at start of follow-up, sex, and for region and urban or rural place of residence. " "Stratified for sex, age (10-yr groups),  and residence" | ≥7cups/day VS ≤2cups/day | 2.11 (0.13, 33.74) | 0.598 |
| **Stensvold. 1994** | Norway | 84/42973 | Melanoma | 10.1 | "With age, cigarettes per day  and county of residence as covariates" | ≥7cups/day VS <2cups/day | 0.45(0.21,0.98) | 0.045- |
| **Veierød.**  **1997** | Norway | 108/50757 | Melanoma | 12.4 | "adjusted for county of residence,  age at inclusion and attained age" | ≥7cups/day VS <2cups/day | 0.61 (0.33, 1.13) | 0.115 |
| **Nilsson.**  **2010** | Sweden | 108/30369 | Melanoma | 6 | age, sex, BMI, smoking, education, and recreational physical activity | ≥4cups/day VS <1cups/day | 0.97 (0.50, 1.89) | 0.939 |
| **Wu.**  **2014** | USA | 363/66484 | Melanoma | 7.7 | age, height, waist–hip ratio, education, income, alcohol, smoking, region of residence, aspirin, and history of nonmelanoma skin cancer." | ≥4cups/day VS <1cups/day | 0.88 (0.64, 1.21) | 0.38 |
| **Loftfield.**  **2015** | USA | 4778/566 398 | Melanoma | 10.5 | Adjusted for age (continuous), sex, cigarette smoking and smoking intensity or current/quit less than one year ago cigar/pipe smoking (ever user or nonuser), body mass index, education, average daily alcohol intake, physical activity, family history of cancer (yes/no), and July erythemal exposure |  | 0.91 (0.81, 1.03) | 0.136 |
| **Lymphoma** | | | | | | | | |
| **Jacobsen.**  **1986** | Norway | 42/16555 | Lymphoma | 11.5 | age at start of follow-up, for sex, and for region and urban or rural place of residence | ≥7cups/day VS ≤2cups/day | 1.58 (0.55, 4.57) | 0.45 |
| **Stensvold. 1994** | Norway | 56/42973 | Hodgkin's/non-  Hodgkin's lymphomas | 10.1 | age, cigarettes per day and county of residence | ≥7cups/day VS <2cups/day | 1.49 (0.45, 4.91) | 0.51 |
| **Nilsson.**  **2010** | Sweden | 111/30369 | Non-Hodgkin’s lymphoma | 6 | age, sex, BMI, smoking, education, and recreational physical activity | ≥4cups/day VS <1cups/day | 1.43 (0.86, 2.38) | 0.849 |

**STable1b: Subgroup analyses of coffee intake and risk of digestive system cancers, meta-regression analysis**

| **Oral, pharyngeal cancer** | | | | |
| --- | --- | --- | --- | --- |
| **Subgroups** | No of  studies | RR  (95%CI) | I2  (%) | P  Heterogeneity |
| **Region** |  |  |  |  |
| **USA** | 2 | 0.69（0.38-1.27） | 91.7 | 0.001 |
| **Europe** | 3 | 0.92（0.67-1.26） | 0 | 0.755 |
| **Asia** | 1 | 0.35（0.16-0.77） | - | - |
| **Follow-up** |  |  |  |  |
| **>10** | 5 | 0.62(0.41-0.94) | 64.3 | 0.024 |
| **<10** | 1 | 0.94(0.73-1.23) | - | - |
| **Adjustment for confounders** | | | | |
| **Smoking** |  |  |  |  |
| **Yes** | 5 | 0.68(0.46-0.99) | 78.5 | 0.001 |
| **No** | 1 | 0.90(0.23-3.50) | - | - |
| **Alcohol** |  |  |  |  |
| **Yes** | 3 | 0.60(0.35-1.01) | 82.6 | 0.001 |
| **No** | 3 | 0.92(0.67-1.26) | 0 | 0.755 |
| **BMI** |  |  |  |  |
| **Yes** | 3 | 0.60(0.35-1.01) | 86.2 | 0.001 |
| **No** | 3 | 0.92(0.67-1.26) | 0 | 0.755 |
| **Physical activity** |  |  |  |  |
| **Yes** | 1 | 1.12(0.93-1.35) | - | - |
| **No** | 5 | 0.62(0.41-0.94) | 64.3 | 0.024 |
| **Total energy** |  |  |  |  |
| **Yes** | 1 | 0.94(0.73-1.23) | - | - |
| **No** | 5 | 0.62(0.41-0.94) | 64.3 | 0.024 |
| **Fiber intake** |  |  |  |  |
| **Yes** | 3 | 0.60(0.35-1.01) | 82.6 | 0.001 |
| **No** | 3 | 0.92(0.67-1.26) | 0 | 0.755 |
| **Esophageal cancer** | | | | |
| **Cancer type**  **Squamous cancer**  **Adenocarcinoma**  **Region** | 3  2 | 0.89 (0.54-1.48)  0.91 (0.66-1.27) | 0.66  0.59 |  |
| **USA** | 1 | 0.95（0.70-1.29） | - | - |
| **Europe** | 3 | 0.89（0.65-1.21） | 0 | 0.693 |
| **Japan** | 2 | 0.66（0.43-1.01） | 0 | 0.412 |
| **Follow-up** |  |  |  |  |
| **>10** | 4 | 0.79(0.61-1.03) | 0 | 0.464 |
| **<10** | 2 | 0.95(0.71-1.27) | 0 | 0.982 |
| **Adjustment for confounders** |  |  |  |  |
| **Smoking** |  |  |  |  |
| **Yes** | 5 | 0.85(0.70-1.03) | 0 | 0.606 |
| **No** | 1 | 1.58(0.35-7.08) | - | - |
| **Alcohol** |  |  |  |  |
| **Yes** | 4 | 0.84(0.68-1.03) | 0 | 0.466 |
| **No** | 2 | 1.05(0.57-1.93) | 0 | 0.558 |
| **BMI** |  |  |  |  |
| **Yes** | 3 | 0.85(0.67-1.08) | 25.1 | 0.263 |
| **No** | 3 | 0.88(0.63-1.21) | 0 | 0.714 |
| **Physical activity** |  |  |  |  |
| **Yes** | 2 | 0.90(0.71-1.14) | 0 | 0.604 |
| **No** | 4 | 0.77(0.54-1.09) | 0 | 0.466 |
| **Total energy** |  |  |  |  |
| **Yes** | 2 | 0.90(0.71-1.14) | 0 | 0.604 |
| **No** | 4 | 0.77(0.54-1.09) | 0 | 0.466 |
| **Fiber intake** |  |  |  |  |
| **Yes** | 3 | 0.83(0.68-1.03) | 19.9 | 0.287 |
| **No** | 3 | 1.02(0.61-1.70) | 0 | 0.827 |
| **Tea intake** |  |  |  |  |
| **Yes** | 3 | 0.76(0.58-1.00) | 0 | 0.492 |
| **No** | 3 | 0.97(0.74-1.28) | 0 | 0.808 |
| **Stomach cancer** | | | | |
| **Sex** |  |  |  |  |
| **Men** | 6 | 1.09(0.90-1.33) | 40.3 | 0.137 0.875 |
| **Women** | 5 | 0.99(0.78-1.24) | 80.9 | 0 |
| **Region** |  |  |  |  |
| **USA** | 3 | 1.36（1.06-1.74） | 0 | 0.574 0.176 |
| **Europe** | 7 | 1.18（0.92-1.51） | 43.4 | 0.101 |
| **Asia** | 2 | 0.87（0.72-1.05） | 0 | 0.54+ |
| **No of cases** |  |  |  |  |
| **<500** | 10 | 1.28(1.09-1.49） | 25.4 | 0.21 0.053 |
| **>500** | 2 | 0.95(0.74-1.21） | 52.5 | 0.147 |
| **Follow-up** |  |  |  |  |
| **>10** | 8 | 1.05(0.92-1.20) | 61.8 | 0.011 0.436 |
| **<10** | 4 | 1.24(1.00-1.54) | 0 | 0.669 |
| **Adjustment for confounders** |  |  |  |  |
| **Smoking** |  |  |  |  |
| **Yes** | 6 | 1.15(0.97-1.36) | 3.2 | 0.396 0.341 |
| **No** | 4 | 1.27(0.85-1.90) | 51.3 | 0.104 |
| **Alcohol** |  |  |  |  |
| **Yes** | 5 | 1.20(0.94-1.53) | 50 | 0.092 0.699 |
| **No** | 5 | 1.20(0.90-1.60) | 11.8 | 0.338 |
| **BMI** |  |  |  |  |
| **Yes** | 4 | 1.06(0.64-1.76) | 78.3 | 0.032 0.18 |
| **No** | 6 | 1.34(1.07-1.66) | 41.2 | 0.131 |
| **Physical activity** |  |  |  |  |
| **Yes** | 4 | 1.12(0.93-1.35) | 0 | 0.454 0.18 |
| **No** | 6 | 1.34(1.07-1.66) | 41.2 | 0.131 |
| **Total energy** |  |  |  |  |
| **Yes** | 2 | 1.18(0.95-1.44) | 0 | 0.391 0.467 |
| **No** | 8 | 1.24(1.01-1.51) | 40.1 | 0.112 |
| **Fiber intake** |  |  |  |  |
| **Yes** | 3 | 1.15(0.96-1.38) | 0 | 0.577 0.344 |
| **No** | 7 | 1.29(1.04-1.61) | 44.5 | 0.094 |
| **Colorectal cancer** | | | | |
| **Cancer types** |  |  |  |  |
| **Colon** | 10 | 0.87(0.78-0.96) | 28.4 | 0.183 0.199 |
| **Rectum** | 10 | 0.94(0.848-1.041) | 0 | 0.942 |
| **Sex** |  |  |  |  |
| **Men** | 7 | 1.07(0.91-1.25) | 0 | 0.583 0.309 |
| **Women** | 8 | 0.92 (0.76-1.12) | 24.1 | 0.237 |
| **Region** |  |  |  |  |
| **USA** | 6 | 0.89（0.81-0.97） | 2.5 | 0.4 0.511 |
| **Europe** | 10 | 1.03（0.95-1.12） | 0 | 0.663 |
| **Asia** | 5 | 0.97（0.82-1.14） | 53 | 0.075 |
| **No of cases** |  |  |  |  |
| **<500** | 10 | 0.90（0.82-0.99） | 9 | 0.36 0.627 |
| **500-1499** | 8 | 1.04（0.92-1.18） | 0 | 0.785 |
| **>1500** | 3 | 0.97（0.9-1.06） | 77.2 | 0.012 |
| **Follow-up** |  |  |  |  |
| **>10** | 11 | 0.98(0.91-1.06) | 37.2 | 0.102 0.633 |
| **<10** | 9 | 0.94(0.86-1.03) | 17.7 | 0.285 |
| **Adjustment for confounders** |  |  |  |  |
| **Alcohol** |  |  |  |  |
| **Yes** | 13 | 0.96(0.9-1.02) | 37.5 | 0.084 0.988 |
| **No** | 8 | 0.98(0.83-1.16) | 0 | 0.435 |
| **Smoking** |  |  |  |  |
| **Yes** | 15 | 0.97(0.91-1.02) | 36.1 | 0.081 0.601 |
| **No** | 6 | 0.93(0.78-1.12) | 0 | 0.524 |
| **BMI** |  |  |  |  |
| **Yes** | 17 | 0.96(0.91-1.02) | 29.9 | 0.118 0.492 |
| **No** | 4 | 0.88(0.64-1.20) | 0 | 0.393 |
| **Physical activity** |  |  |  |  |
| **Yes** | 13 | 0.98(0.91-1.05) | 42.6 | 0.052 0.536 |
| **No** | 8 | 0.93(0.84-1.02) | 0 | 0.726 |
| **Red meat** |  |  |  |  |
| **Yes** | 6 | 0.95(0.88-1.02) | 44.2 | 0.11 0.84 |
| **No** | 15 | 0.95(0.88-1.03) | 18 | 0.252 |
| **Total energy** |  |  |  |  |
| **Yes** | 7 | 0.96(0.88-1.03) | 52.9 | 0.048 0.631 |
| **No** | 14 | 0.97(0.89-1.05) | 3.1 | 0.416 |
| **Fiber intake** |  |  |  |  |
| **Yes** | 9 | 0.98(0.91-1.06) | 16.3 | 0.297 0.483 |
| **No** | 12 | 0.93(0.85-1.02) | 30 | 0.152 |
| **Family history** |  |  |  |  |
| **Yes** | 9 | 0.95(0.86-1.04) | 28.4 | 0.192 0.565 |
| **No** | 12 | 0.97(0.9-1.04) | 25.8 | 0.190 |
| **Tea intake** |  |  |  |  |
| **Yes** | 5 | 0.91(0.77-1.07) | 0 | 0.483 0.647 |
| **No** | 16 | 0.97(0.91-1.03) | 32.4 | 0.103 |
| **Diabetes** |  |  |  |  |
| **Yes** | 6 | 0.98(0.91-1.06） | 45.7 | 0.101 0.625 |
| **No** | 15 | 0.94(0.87-1.02) | 15.3 | 0.282 |
| **Pancreatic cancer** | | | | |
| **Sex** |  |  |  |  |
| **Men** | 8 | 0.95(0.72-1.26) | 0 | 0.543 0.252 |
| **Women** | 5 | 1.43(0.89-2.30) | 13.3 | 0.329 |
| **Region** |  |  |  |  |
| **USA** | 5 | 1.11（0.79-1.54） | 35.4 | 0.185 0.551 |
| **Europe** | 7 | 0.99（0.83-1.19） | 16 | 0.308 |
| **Asia** | 3 | 1.02（0.6-1.71） | 34.1 | 0.219 |
| **Follow-up** |  |  |  |  |
| **>10** | 8 | 0.95(0.8-1.13) | 20.9 | 0.264 0.069 |
| **<10** | 7 | 1.46(0.98-2.16) | 0 | 0.665 |
| **Adjustment for confounders** |  |  |  |  |
| **Smoking** |  |  |  |  |
| **Yes** | 13 | 1.01 (0.86-1.19) | 27.4 | 0.168 0.826 |
| **No** | 2 | 1.06(0.67-1.68) | 0 | 0.727 |
| **Alcohol** |  |  |  |  |
| **Yes** | 2 | 0.81(0.51-1.29) | 16.5 | 0.269 0.383 |
| **No** | 13 | 1.04(0.89-1.23) | 23.3 | 0.208 |
| **BMI** |  |  |  |  |
| **Yes** | 5 | 0.98(0.87-1.17) | 0 | 0.595 0.595 |
| **No** | 10 | 1.09(0.84-1.42) | 33.2 | 0.142 |
| **Diabetes** |  |  |  |  |
| **Yes** | 4 | 0.96(0.8-1.16) | 0 | 0.571 0.403 |
| **No** | 11 | 1.12(0.87-1.44) | 27.8 | 0.18 |
| **History of cholelithiasis** |  |  |  |  |
| **Yes** | 2 | 0.73(0.45-1.19) | 16.5 | 0.269 0.226 |
| **No** | 13 | 1.05(0.9-1.24) | 17.4 | 0.269 |
| **Residence** |  |  |  |  |
| **Yes** | 3 | 0.97(0.60-1.58) | 0 | 0.269 0.871 |
| **No** | 12 | 1.02(0.87-1.20) | 26.6 | 0.183 |
| **Liver cancer** | | | | |
| **Sex** |  |  |  |  |
| **Men** | 3 | 0.29(0.16-0.50) | 0 | 0.977 |
| **Women** | 3 | 0.68(0.31-1.46) | 0 | 0.692 |
| **Region** |  |  |  |  |
| **USA** | 1 | 0.59（0.35-0.99） | - | - |
| **Europe** | 3 | 0.37（0.26-0.52） | 22.6 | 0.275 |
| **Asia** | 5 | 0.51（0.37-0.70） | 0 | 0.673 |
| **Follow-up** |  |  |  |  |
| **>10** | 8 | 0.46(0.36-0.59) | 0 | 0.431 |
| **<10** | 1 | 0.58(0.36-0.95) | - | - |
| **Adjustment for confounders** |  |  |  |  |
| **BMI** |  |  |  |  |
| **Yes** | 7 | 0.45(0.35-0.57) | 0 | 0.454 |
| **No** | 2 | 0.51(0.32-0.79) | 49.1 | 0.161 |
| **Tea intake** |  |  |  |  |
| **Yes** | 4 | 0.50(0.35-0.72) | 0 | 0.610 |
| **No** | 5 | 0.44(0.34-0.58) | 30.4 | 0.219 |
| **Diabetes** |  |  |  |  |
| **Yes** | 7 | 0.45(0.35-0.57) | 0 | 0.454 |
| **No** | 2 | 0.51(0.32-0.79) | 49.1 | 0.161 |
| **Liver disease** |  |  |  |  |
| **Yes** | 4 | 0.36(0.23-0.56) | 0 | 0.714 |
| **No** | 5 | 0.50(0.39-0.64) | 18.4 | 0.297 |

**Stable2b: Subgroup analyses of coffee intake and risk of urinary system cancer, meta-regression analysis**

| **Renal cancer** | | | | |
| --- | --- | --- | --- | --- |
| **Subgroups** | No of  studies | RR  (95%CI) | I2  (%) | P  Heterogeneity |
| **Region** |  |  |  |  |
| **USA** | 1 | 0.87(0.62-1.22) | - | - |
| **Europe** | 4 | 0.65(0.33-1.30) | 62.1 | 0.048 |
| **Follow-up** |  |  |  |  |
| **>10** | 3 | 0.84(0.61-1.15) | 0.6 | 0.366 |
| **<10** | 2 | 0.62(0.18-2.07) | 82.4 | 0.017 |
| **Adjustment for confounders** | | | | |
| **Smoking** |  |  |  |  |
| **Yes** | 4 | 0.85(0.54-1.22) | 48.7 | 0.119 |
| **No** | 1 | 0.30(0.07-1.33) | - | - |
| **Physical activity** |  |  |  |  |
| **Yes** | 1 | 0.30(0.11-0.80) | - | - |
| **No** | 4 | 0.94(0.76-1.17) | 0.3 | 0.39 |
| **BMI** |  |  |  |  |
| **Yes** | 3 | 0.81(0.53-1.25) | 65.6 | 0.055 |
| **No** | 2 | 0.63(0.20-2.00) | 43.9 | 0.182 |
| **Residence** |  |  |  |  |
| **Yes** | 1 | 1.01(0.38-2.67) | - | - |
| **No** | 4 | 0.92(0.67-1.26) | 62.1 | 0.048 |
| **Hypertension** |  |  |  |  |
| **Yes** | 1 | 0.87(0.62-1.11) | - | - |
| **No** | 4 | 0.65(0.33-1.30) | 62.1 | 0.048 |
| **Bladder cancer** | | | | |
| **Sex** |  |  |  |  |
| **Men** | 5 | 1.30(0.89-1.88) | 42.5 | 0.138 0.967 |
| **Women** | 4 | 0.84(0.34-2.06) | 78.3 | 0.003 |
| **Region** |  |  |  |  |
| **USA** | 5 | 1.38(1.02-1.85） | 52 | 0.08 0.455 |
| **Europe** | 3 | 1.03(0.78-1.35） | 44.1 | 0.167 |
| **Asia** | 2 | 0.97(0.67-1.40） | 0 | 0.737 |
| **Follow-up** |  |  |  |  |
| **>10** | 8 | 1.17(0.94-1.46) | 40.3 | 0.11 0.826 |
| **<10** | 2 | 1.05(0.79-1.41) | 65.3 | 0.09 |
| **Adjustment for confounders** |  |  |  |  |
| **Smoking** |  |  |  |  |
| **Yes** | 7 | 1.05(0.86-1.28) | 38.8 | 0.133 0.23 |
| **No** | 3 | 1.45(0.99-2.12) | 31.2 | 0.234 |
| **Tea intake** |  |  |  |  |
| **Yes** | 3 | 0.93(0.73-1.17) | 0 | 0.752 0.034 |
| **No** | 7 | 1.44(1.10-1.89) | 28.8 | 0.209 |
| **Alcohol** |  |  |  |  |
| **Yes** | 1 | 1.02(0.62-1.68) | - | - 0.4 |
| **No** | 9 | 1.14(0.94-1.38) | 45.7 | 0.064 |
| **BMI** |  |  |  |  |
| **Yes** | 1 | 0.90(0.52-1.56) | - | - 0.274 |
| **No** | 9 | 1.15(0.96-1.39) | 43.7 | 0.077 |
| **Fiber intake** |  |  |  |  |
| **Yes** | 1 | 0.79(0.48-1.30) | - | - 0.141 |
| **No** | 9 | 1.18(0.98-1.43) | 37 | 0.123 |
| **Prostate cancer** | | | | |
| **Region** |  |  |  |  |
| **USA** | 5 | 0.93(0.87-1.00) | 11.1 | 0.343 0.198 |
| **Europe** | 6 | 0.81(0.74-0.89) | 0 | 0.565 |
| **Canada** | 1 | 1.42(0.77-2.61) | - | - |
| **Asia** | 2 | 0.85(0.64-1.141) | 60.6 | 0.111 |
| **Follow-up** |  |  |  |  |
| **>10** | 12 | 0.88(0.83-0.93) | 33.7 | 0.121 0.379 |
| **<10** | 2 | 1.05(0.82-1.34) | 0 | 0.808 |
| **Adjustment for confounders** |  |  |  |  |
| **Smoking** |  |  |  |  |
| **Yes** | 5 | 0.81(0.73-0.89) | 2.1 | 0.394 0.669 |
| **No** | 9 | 0.92(0.86-0.99) | 16.3 | 0.297 |
| **Alcohol** |  |  |  |  |
| **Yes** | 4 | 0.84(0.75-0.94) | 0 | 0.394 0.658 |
| **No** | 10 | 0.90(0.85-0.96) | 37.7 | 0.108 |
| **Physical activity** |  |  |  |  |
| **Yes** | 5 | 0.88(0.83-0.93) | 53.3 | 0.073 0.843 |
| **No** | 9 | 0.95(0.80-1.13) | 15 | 0.309 |
| **BMI** |  |  |  |  |
| **Yes** | 7 | 0.87(0.83-0.93) | 42.7 | 0.106 0.390 |
| **No** | 7 | 0.63(0.20-2.00) | 0.3 | 0.421 |
| **Diabetes** |  |  |  |  |
| **Yes** | 2 | 0.80(0.73-0.88) | 7 | 0.377 0.376 |
| **No** | 12 | 0.93(0.87-0.99) | 0 | 0.565 |
| **Family history** |  |  |  |  |
| **Yes** | 4 | 0.89(0.84-0.95) | 47.6 | 0.126 0.981 |
| **No** | 10 | 0.87(0.78-0.96) | 28.8 | 0.179 |
| **Total energy** |  |  |  |  |
| **Yes** | 3 | 0.90(0.84-0.96) | 44.1 | 0.135 0.577 |
| **No** | 9 | 0.85(0.77-0.94) | 30.2 | 0.159 |
| **PSA testing** |  |  |  |  |
| **Yes** | 2 | 0.92(0.86-0.99) | 44.6 | 0.179 0.244 |
| **No** | 12 | 0.84(0.78-0.92) | 24 | 0.208 |

**Stable3b: Subgroup analyses of coffee intake and female genital cancers, meta-regression** analysis

| **Breast cancer** | | | | |
| --- | --- | --- | --- | --- |
| **Subgroups** | No of  studies | RR  (95%CI) | I2  (%) | P  Heterogeneity |
| **Region** |  |  |  |  |
| **USA** | 6 | 0.97（0.91-1.03） | 0 | 0.739 |
| **Europe** | 8 | 1.00（0.93-1.09） | 20.8 | 0.264 |
| **Asia** | 3 | 1.08（0.90-1.29） | 0 | 0.434 |
| **No of cases** |  |  |  |  |
| **<500** | 6 | 1.09（0.94-1.28） | 45 | 0.106 |
| **500-1499** | 7 | 0.98（0.90-1.07） | 0 | 0.917 |
| **>1500** | 4 | 0.98（0.92-1.04） | 0 | 0.617 |
| **Follow-up** |  |  |  |  |
| **>10** | 9 | 0.97(0.92-1.04) | 14.7 | 0.311 |
| **<10** | 8 | 1.01(0.94-1.08) | 0 | 0.672 |
| **Adjustment for confounders** | | | | |
| **Alcohol** |  |  |  |  |
| **Yes** | 7 | 0.96(0.91-1.02) | 0 | 0.547 |
| **No** | 10 | 1.04(0.96-1.13) | 13.3 | 0.321 |
| **Smoking** |  |  |  |  |
| **Yes** | 7 | 0.97(0.91-1.03) | 0 | 0.818 |
| **No** | 10 | 1.02(0.95-1.1) | 14.1 | 0.313 |
| **BMI** |  |  |  |  |
| **Yes** | 11 | 1.09(0.94-1.28) | 45 | 0.106 |
| **No** | 6 | 0.98(0.93-1.03) | 0 | 0.955 |
| **Physical activity** |  |  |  |  |
| **Yes** | 6 | 0.95(0.88-1.03) | 0 | 0.775 |
| **No** | 11 | 1.01(0.95-1.07) | 7.7 | 0.371 |
| **Age at menopause** |  |  |  |  |
| **Yes** | 8 | 0.98(0.93-1.03) | 0 | 0.829 |
| **No** | 9 | 1.01(0.91-1.12) | 26.6 | 0.208 |
| **Total energy** |  |  |  |  |
| **Yes** | 8 | 0.99(0.94-1.05) | 0 | 0.95 |
| **No** | 9 | 0.98(0.90-1.06) | 35.7 | 0.133 |
| **Use of oral contraception** |  |  |  |  |
| **Yes** | 6 | 0.97(0.91-1.03) | 0 | 0.791 |
| **No** | 11 | 1.01(0.94-1.08) | 13.4 | 0.317 |
| **Family history** |  |  |  |  |
| **Yes** | 10 | 0.98(0.93-1.04) | 0 | 0.921 |
| **No** | 7 | 1.00(0.93-1.08) | 43.9 | 0.098 |
| **Postmenopausal hormones** |  |  |  |  |
| **Yes** | 6 | 0.99(0.93-1.04) | 0 | 0.723 |
| **No** | 11 | 0.99(0.91-1.08) | 15.5 | 0.296 |
| **History of benign**  **breast disease** |  |  |  |  |
| **Yes** | 4 | 0.97（0.91-1.04） | 0 | 0.394 |
| **No** | 13 | 1.00(0.94-1.06) | 0 | 0.498 |
|  |  | **Ovarian cancer** |  |  |
| **Region** |  |  |  |  |
| **USA** | 2 | 0.86（0.68-1.10） | 64.2 | 0.094 |
| **Europe** | 5 | 1.10（0.90-1.34） | 0 | 0.700 |
| **Canada** | 1 | 1.62（0.95-1.34） | - | - |
| **No of cases** |  |  |  |  |
| **<500** | 5 | 1.20（0.96-1.49） | 0 | 0.613 |
| **>500** | 3 | 0.92（0.76-112） | 45.7 | 0.158 |
| **Adjustment for confounders** |  |  |  |  |
| **Smoking** |  |  |  |  |
| **Yes** | 7 | 1.03 (0.88-1.21) | 41.9 | 0.112 |
| **No** | 1 | 1.04(0.73-1.50) | — | — |
| **Alcohol** |  |  |  |  |
| **Yes** | 2 | 1.19(0.89-1.57) | 45.5 | 0.175 |
| **No** | 6 | 0.98(0.83-1.17) | 31.1 | 0.202 |
| **BMI** |  |  |  |  |
| **Yes** | 5 | 1.00(0.83-1.19) | 50 | 0092 |
| **No** | 3 | 1.11(0.87-1.42) | 0 | 0.393 |
| **Physical activity** |  |  |  |  |
| **Yes** | 4 | 0.98(0.79-1.21) | 61.9 | 0.049 |
| **No** | 4 | 1.04(0.89-1.20) | 0 | 0.586 |
| **Menopausal status** |  |  |  |  |
| **Yes** | 3 | 1.21(0.94-1.55) | 0 | 0.387 |
| **No** | 5 | 0.95(0.80-1.14) | 35.2 | 0.187 |
| **Oral contraceptive** |  |  |  |  |
| **Yes** | 6 | 1.01(0.87-1.17) | 35.3 | 0.172 |
| **No** | 2 | 1.74(0.87-3.49) | 0 | 0.548 |
| **Energy intake** |  |  |  |  |
| **Yes** | 4 | 1.15(0.94-1.41) | 0 | 0.508 |
| **No** | 4 | 0.93(0.75-1.14) | 48.7 | 0.119 |
| **Parity** |  |  |  |  |
| **Yes** | 5 | 1.13(0.95-1.36) | 0 | 0.66 |
| **No** | 3 | 0.86(0.67-1.11) | 58.9 | 0.088 |
| **Endometrial cancer** | | | | |
| **Region** |  |  |  |  |
| **USA** | 4 | 0.72（0.67-0.80） | 0 | 0.498 |
| **Europe** | 7 | 0.75（0.66-0.85） | 0 | 0.58 |
| **Japan** | 1 | 0.38（0.16-0.91） | - | - |
| **Follow up** |  |  |  |  |
| **<10** | 4 | 0.71(0.60-0.84) | 0 | 0.427 |
| **>10** | 8 | 0.74(0.66-0.83) | 0 | 0.484 |
| **Adjustment for confounders** |  |  |  |  |
| **Smoking** |  |  |  |  |
| **Yes** | 11 | 0.73 (0.67-0.80) | 0 | 0.533 |
| **No** | 1 | 0.35(0.04-2.93) | - | - |
| **Alcohol** |  |  |  |  |
| **Yes** | 2 | 0.74(0.60-0.90) | 0 | 0.866 |
| **No** | 10 | 0.73(0.66-0.81) | 4.3 | 0.4 |
| **BMI** |  |  |  |  |
| **Yes** | 10 | 0.73(0.67-0.80) | 0 | 0.442 |
| **No** | 2 | 0.71(0.32-1.58) | 0 | 0.482 |
| **Physical activity** |  |  |  |  |
| **Yes** | 2 | 0.66(0.53-0.82) | 0 | 0.395 |
| **No** | 10 | 0.75(0.67-0.83) | 0 | 0.570 |
| **Age at menarche** |  |  |  |  |
| **Yes** | 5 | 0.74(0.68-0.82) | 0 | 0.371 |
| **No** | 7 | 0.69(0.57-0.85) | 7.4 | 0.371 |
| **Menopausal status** |  |  |  |  |
| **Yes** | 7 | 0.73(0.66-0.81) | 0 | 0.525 |
| **No** | 5 | 0.73(0.59-0.92) | 7.1 | 0.366 |
| **Oral contraceptive** |  |  |  |  |
| **Yes** | 5 | 0.73(0.65-0.81) | 22.9 | 0.269 |
| **No** | 7 | 0.74(0.62-0.90) | 0 | 0.647 |
| **Energy intake** |  |  |  |  |
| **Yes** | 3 | 0.74(0.63-0.87) | 0 | 0.982 |
| **No** | 9 | 0.72(0.65-0.81) | 14.5 | 0.313 |
| **Parity** |  |  |  |  |
| **Yes** | 6 | 0.72(0.65-0.80) | 7.7 | 0.367 |
| **No** | 6 | 0.76(0.62-0.93) | 0 | 0.575 |
| **History of diabetes** |  |  |  |  |
| **Yes** | 4 | 0.69(0.59-0.79) | 0 | 0.809 |
| **No** | 8 | 0.76(0.68-0.86) | 4.7 | 0.394 |

**Stable4b: Subgroup analyses of coffee intake and risk of other cancers, meta-regression** analysis

| **Lung cancer** | | | | |
| --- | --- | --- | --- | --- |
| **Subgroups** | No of  studies | RR  (95%CI) | I2  (%) | P  Heterogeneity |
| **Sex** |  |  |  |  |
| **Men** | 2 | 3.33(1.34-7.92) | 54.9 | 0.136 0.816 |
| **Women** | 1 | 1.72(1.16-2.56) | — | — |
| **Region** |  |  |  |  |
| **USA** | 1 | 2.33（1.25-4.32） | — | — 0.331 |
| **Europe** | 2 | 3.06（0.92-10.13） | 81.5 | 0.02 |
| **Japan** | 1 | 1.20（0.60-2.40） | — | — |
| **Adjustment for confounders** | | | | |
| **Smoking** |  |  |  |  |
| **Yes** | 3 | 2.45(1.07-5.59) | 73.6 | 0.022 0.894 |
| **No** | 1 | 1.76(1.07-2.89) | - | - |
| **Melanoma** | | | | |
| **Region** |  |  |  |  |
| **USA** | 2 | 0.91（0.72-1.14） | 0 | 0.782 |
| **Europe** | 4 | 0.86（0.51-1.47） | 0 | 0.771 |
| **Follow-up** |  |  |  |  |
| **>10** | 4 | 0.91(0.67-1.23) | 0 | 0.763 |
| **<10** | 2 | 0.90(0.67-1.19) | 0 | 0.796 |
| **Adjustment for confounders** |  |  |  |  |
| **Smoking** |  |  |  |  |
| **Yes** | 4 | 0.90 (0.73-1.11) | 0 | 0.859 |
| **No** | 2 | 0.88(0.27-2.82) | 0 | 0.493 |
| **Alcohol** |  |  |  |  |
| **Yes** | 2 | 0.91(0.72-1.14) | 0 | 0.782 |
| **No** | 4 | 0.86(0.51-1.47) | 0 | 0.771 |
| **BMI** |  |  |  |  |
| **Yes** | 2 | 0.94(0.71-1.26) | 0 | 0.929 |
| **No** | 4 | 0.86(0.64-1.16) | 0 | 0.797 |
| **Physical activity** |  |  |  |  |
| **Yes** | 2 | 0.94(0.71-1.26) | 0 | 0.929 |
| **No** | 4 | 0.86(0.64-1.16) | 0 | 0.797 |
